# Identification of key regulators in glycogen utilization in E. coli based on the simulations from a hybrid functional Petri net model

**DOI:** 10.1186/1752-0509-7-S6-S1

**Published:** 2013-12-13

**Authors:** Zhongyuan Tian, Adrien Fauré, Hirotada Mori, Hiroshi Matsuno

**Affiliations:** 1 Graduate School of Science and Engineering, Yamaguchi University, 1677-1 Yoshida, 753-8512 Yamaguchi-shi, Yamaguchi, Japan; 2 Graduate School of Biological Sciences, Nara Institute of Science and Technology, 8916-5 Takayama, 630-0101 Ikoma, Nara, Japan

## Abstract

**Background:**

Glycogen and glucose are two sugar sources available during the lag phase of *E. coli*, but the mechanism that regulates their utilization is still unclear.

**Methods:**

Attempting to unveil the relationship between glucose and glycogen, we propose an integrated hybrid functional Petri net (HFPN) model including glycolysis, PTS, glycogen metabolic pathway, and their internal regulatory systems.

**Results and conclusions:**

By comparing known biological results to this model, basic necessary regulatory mechanism for utilizing glucose and glycogen were identified as a feedback circuit in which HPr and EIIA*^Glc ^*play key roles. Based on this regulatory HFPN model, we discuss the process of glycogen utilization in *E. coli *in the context of a systematic understanding of carbohydrate metabolism.

## Background

The carbohydrate pathway occupies a central position in a cell's metabolism. In our previous paper [1], we proved that glycogen plays an important role in the lag phase of *E. coli*. But how the cell regulates the utilization of these carbon sources, intracellular glycogen and extracellular glucose, was yet to be clarified. In a cell, glycogen works as a sugar store or a sugar supply depending on different nutrition conditions, under the regulation of enzymes expressed by *glg *gene clusters (*glgBXCAP*) [2]. Uptake of extracellular glucose is conducted via the phosphotransferase system (PTS) in *E. coli*, whose enzymes are expressed from two operons, *ptsHIcrr *and *ptsG *[3]. Although several shared regulators of PTS and glycogen metabolism, such as ppGpp, Cra, CsrA and cAMP/CRP, have been studied [2,4-10], a basic regulation system for the utilization of glucose and glycogen has not been studied yet.

Computer modeling is a general and effective method for the integration of biological systems. In our previous work [1], at first we calculated glucose and G6P concentrations, from which we predicted the existence of another major sugar donor, glycogen, in the lag phase. The function of glycogen as a sugar donor was simulated, and demonstrated experimentally. The purpose of this paper is to construct an integrated model for the systematic understanding of the carbohydrate pathway system of *E. coli*. In this work we firstly constructed two hybrid functional Petri net (HFPN) models [11] based on two types of published models: ordinary differential equation (ODE) model of central carbohydrate pathway [12] and a mass balance theory model of PTS [13]. These two models were then assembled together with a newly developed general mass action model of the glycogen metabolic pathway into a single, comprehensive HFPN model.

By applying metabolic regulatory mechanisms in our combined HFPN model, a basic control system regulating the utilization glucose and glycogen was identified, in which HPr::GlgP complex [14-16], EIIA*^Glc^*&cAMP system [8,17], EI dimerization [18,19], FDP&Cra mutual feedback [6], HPr subcellular location [2,16,20] etc. are working as regulators. In this paper, with the support of simulation results from the HFPN model, we clarify functions of HPr and EIIA*^Glc ^*as key regulators of glucose and glycogen utilization.

## Results

### Molecular mechanisms for regulating glucose and glycogen utilization

**Figure 1:**
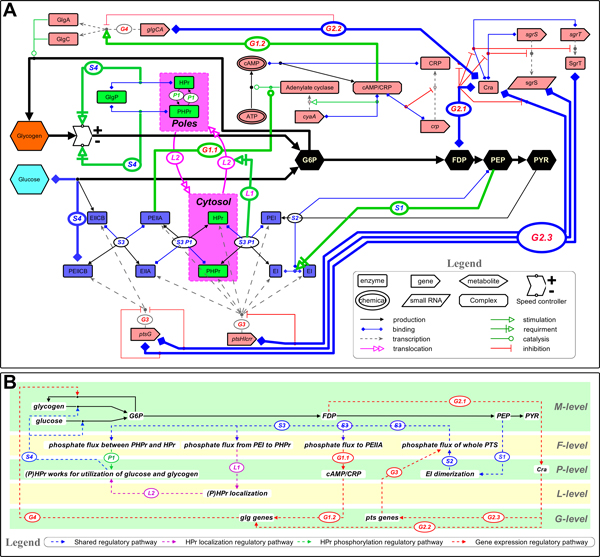
**Basic regulation mechanisms required in the process of a cell utilizing glucose and glycogen**. **A **molecular interaction map [21] of basic regulators that control glucose and glycogen utilization. **B **hierarchical map of **A**. Edge labels are shared in **A **and **B**. **Px **marked edges (x means number, e.g. **P1**) denote regulations only belong to HPr phosphorylation regulatory pathway; **Lx **marked regulations (e.g. **L1**) are only belong to HPr localization regulatory pathway; **Gx **marked regulations (e.g. **G1.1**) are only of gene expression regulatory pathway; and **Sx **marked regulations (e.g. **S3**) are of shared regulatory pathway, which is shared by aforementioned 3 pathways.

There are many regulators working on central metabolism of *E. coli *[2,4-10]. Among them we selected basic regulators that control glucose and glycogen utilization, which is illustrated in Figure 1A using the molecular interaction map notation [21], a widely recognized standard notation capable of describing biological regulatory networks in a way of electronic circuit. These basic regulators constitute a circuit that gives a whole view of the regulation of glucose and glycogen utilization as showing in Figure 1B. These components are classified into 3 regulation pathways (HPr phosphorylation regulatory pathway (includes actions labeled ***S1***, ***S2***, ***S3***, ***P1***, ***S4)***, HPr localization regulatory pathway (includes actions labeled ***S1***, ***S2***, ***S3***, ***L1***, ***L2***, ***S4)***, and gene expression regulatory pathway in **PEIIA***^Glc^***&cAMP **subpath-way (includes actions labeled ***S1***, ***S2***, ***S3***, ***G1.1***, ***G1.2***, ***G3)***, and in **FDP&Cra **subpathway (includes actions labeled ***G2.1***, ***G2.2***, ***G3***, ***G4)***) "Shared regulatory pathway" denotes shared pathways of aforementioned 3 pathways. This diagram is constituted by 5 level hierarchies of metabolite level (M-level), molecule localization level (L-level), phosphate flux level (F-level), protein level(P-level), gene expression level (G-level).

#### HPr phosphorylation regulatory pathway

Transition of phosphorylation states of HPr is in charge of the functions of PTS and GlgP [2,8], which occupying a key role at regulation of glucose and glycogen utilization. This HPr centered self feedback control pathway starts from PEP concentration (M-level), terminates at glycogen decomposition (M-level).

Regulations in this pathway includes actions labeled ***S1***, ***S2***, ***S3***, ***P1***, ***S4***. Regulation ***S1 ***(from M-level to P-level): high enough PEP levels activate the phosphate group influx into PTS by stimulating EI dimerization [18,19]. Regulation ***S2 ***(from P-level to F-level): EI dimerization controls phosphate influx into PTS, which is thought to be the limiting step in the process of phosphate group transfering from PEP to G6P via PTS [19]. Regulation ***S3 ***(from F-level to F-level): Different phosphorylation states of HPr is resulted from the balance of phosphate group influx to PTS from PEP and outflux to G6P from PTS. Regulation ***P1 ***(from F-level to P-level): Different phosphorylation states of HPr result in different phosphorylation states of protein to protein interactions (P)HPr::GlgP (From here on, PHPr denotes the phosphorylated form of HPr, HPr denotes the unphosphorylated form, and (P)HPr denotes both phosphorylated and unphosphorylated Hpr), or (P)EIIA*^Glc^*::(P)HPr::(P)EI (HPr works as a member enzyme of PTS). Regulation ***S4 ***(from P-level to M-level): The phosphorylation state of the (P)HPr::GlgP complex controls glycogen decomposition, of which catalyzing speed of HPr::GlgP is about five times higher than that of PHPr::GlgP [14]. In PTS, HPr transfers phosphate group from PEI to EIIA*^Glc^*. Thus (P)HPr regulates the speed of carbohydrate decompositions from both glycogen and glucose.

#### HPr localization regulatory pathway

Lopian et al. (2010) described the spatial and temporal organization of PTS enzymes in *E. coli*, especially HPr and EI [20]. According to their study, HPr and EI mainly stay in the poles of a cell independently, and if HPr is released to the cytosol, it should be phosphorylated by PEI in the presence of glucose. Genobase also supplies a GFP photo of HPr localization, which is scattered in the cytosol [22]. In the glycogen metabolism, interestingly, glycogenesis enzymes (GlgC, GlgA) and glycogen granules locate at the poles, while GlgP is scattered in the cytosol [2]. GlgP is considered to be always bound in a complex with HPr, since the concentration of HPr is much higher than that of GlgP in *E. coli *[14,15].

Based on these studies, we hypothesize that HPr controls the priority in glucose and glycogen utilization in *E. coli*. (1) If there is no glucose, HPr cannot get phosphate from EI, keeping its location at the poles. Hence, this pole-located HPr mainly serves for glycogen decomposition, whose speed is regulated by phosphorylation state of (P)HPr::GlgP. (2) If there is a little glucose supply, at the very beginning of lag phase, glucose uptake takes place at poles areas for a very short time until all the phosphates are removed from these PTS enzymes, including HPr. Note that the pole-located HPr also has the ability of exchanging phosphate with other PTS enzymes. (3) If glucose is abundant, HPr gets phosphate group from PEI, causing its release to the cytosol. Cytosol-scattered HPr works as a PTS protein, but not for glycogenolysis, hence, transporting phosphate from EI to EIIA*^Glc^*.

Regulations in this pathway includes actions labeled ***S1***, ***S2***, ***S3***, ***L1***, ***L2***, ***S4***. Regulations ***S1***, ***S2***, and ***S4 ***are the same as those in phosphate flux regulatory pathway. Regulation ***S3 ***(from F-level to F-level): The flux of phosphate group into PTS from PEP influences the flux of phosphate group from PEI to PHPr. Regulation ***L1 ***(from F-level to L-level): When there are phosphate groups flux from PEI to PHPr, PHPr will be translocated from poles to the whole cytosol [20]. Regulation ***L2 ***(from L-level to P-level): When (P)HPr is located at the cell's poles, it mainly functions for glycogen phosphorylation. And when (P)HPr is scattered in cytosol, it serves for the function of PTS, which is responsible for glucose uptake. At last this pathway goes back to regulation ***S4 ***to finish its regulation.

#### Gene expression regulatory pathway

This pathway explains how an *E. coli *controls glycogen and glucose utilization in gene expression level. PTS enzymes for glucose uptake in *E. coli *include EI, HPr, EIIA*^Glc ^*and EIICB*^Glc^*, in which the former three enzymes are expressed from *ptsHI-crr *gene cluster and EIICB*^Glc ^*is from *ptsG*. Cra is known as a global DNA-binding regulator of the genes for carbon metabolism in *E. coli*, which directly regulates *ptsHIcrr *operon [6], and indirectly influences *ptsG *transcription via SgrT and small RNA SgrS pathway [6,23]. Fructose-1,6-bisphosphate (FDP) inhibits free Cra level by binding its gene [6]. In *E. coli*, clustering methods of glycogen associated genes are complex and discussable, such as *glgBXCAP, glgBX, glgCAP, glgAP *etc. [2,17,24], in which GlgC and GlgA catalyzed glycogen synthesis have been experimentally proved to be regulated by cAMP/CRP and Cra [6,17]. Gene expression regulatory pathway of this study includes two subpathways, PEIIA*^Glc^*&cAMP subpathway and FDP&Cra subpathway.

In **PEIIA***^Glc^***&cAMP **subpathway (includes actions labeled ***S1***, ***S2***, ***S3***, ***G1.1***, ***G1.2***, ***G3***), regulations ***S1 ***and ***S2 ***are shared with the other two regulation path-ways. Regulation ***S3 ***(from F-level to F-level): The flux of phosphate group into PTS from PEP influences the phosphate intraflux between EIIA*^Glc ^*and PEIIA*^Glc^*. Regulation ***G1.1 ***(F-level to P-level): The phosphate flux between EIIA*^Glc ^*and PEIIA*^Glc ^*controls intracellular PEIIA*^Glc ^*concentration, which stimulates adenylate cyclase (AC) to produce much cAMP. In Figure 1A, we can see a local feedback loop constituted by cAMP, CRP and cAMP/CRP, the binding complex of them [7,8,17]. Regulation ***G1.2 ***(P-level to G-level): cAMP/CRP network upregulating *glgC *and *glgA *expression is confirmed by experiments of [17]. Because expressions of *glgC *and *glgA *(Regulation ***G4***) are under combined regulation of PEIIA*^Glc^*&cAMP subpathway and FDP&Cra subpathway, we will explain it later in the following subpathway.

In **FDP&Cra **subpathway (includes actions labeled ***G2.1***, ***G2.2***, ***G3***, ***G4***), regulations ***G2.1 ***(M-level to P-level) and ***G2.2 ***(P-level to G-level): When FDP reaches a high level, Cra expression is repressed, which releases its regulations on *glgC, glgA, ptsHIcrr *directly and *ptsG *via SgrST route [6]. Regulation ***G3 ***(G-level to F-level): After an exponential increasing, when an enzyme concentration increases above a certain threshold, its catalyzed reaction speed will remain in a high level [25]. Here we assumed that, when PTS enzymes are expressed above a certain threshold, the whole PTS reaction speed would be extremely accelerated. Regulation ***G4 ***(G-level to M-level): *glgC *and *glgA *expression levels are under regulations from both PEIIA*^Glc^*&cAMP subpathway and FDP&Cra subpathway. Comprehensively say, when Cra levels decreases, it releases the inhibition of *glgC *and *glgA*, as a consequence cAMP/CRP activates extremely strong expression of *glgC *and *glgA *(Details are discussed in Results section).

Construction of a dynamic simulation model of central metabolic pathway with HFPN Central metabolic pathway in *E. coli *is constituted by the glycolysis, the pentose phosphate (PP) pathway, and the tricarboxylic acid cycle (TCA cycle). Most glycolysis models are based on ODE [12,26,27]. Chassagnole et al. (2002) constructed an integrated ODE model of glycolysis and PP pathways [12,28], which is often used as a base model in many studies [26,27,29,30]. By assembling TCA cycle with the model of [12], Kadir et al. (2010) set up an ODE model together with six pieces of logical controlling rules [27], and Usuda et al. (2010) included gene regulation in [26]. Kinetic parameters of these ODE model were taken from the databases, such as BRENDA [31], SABIO-RK [32], and BioModels [33], and some works focused on parameter optimization [30]. PTS are usually represented by one or a few equations in these ODE models. Rohwer at el (2000) set a mass balance theory model of PTS, by using experimentally tested mass action constant for each elementary biochemical reaction within PTS enzymes [13], and some studies are based on it [9,34].

**Figure 2:**
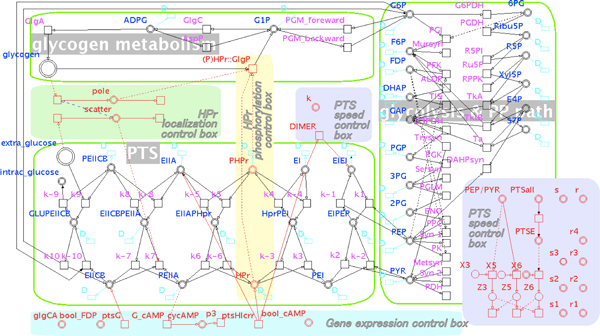
**A HFPN model of extended center metabolism pathway in E. coli**. This model includes three major parts: glycolysis and pentose phosphate pathway part, PTS part and glycogen metabolism pathway part, and they are bordered by **light green lines **respectively. **Cyan border and D marked transitions **are degradation or dilution processes of their connected metabolites or enzymes. **Black border components **are of the three major parts. **Red border components **are of regulatory mechanisms, in which transition *ptsG *and *ptsHIcrr *represent PTS genes expression process; **G_cAMP **is the process of PEIIA*^Glc ^*activating cAMP production; **Dimer **is the process of EI dimerization; **k **is the parameter controlling the whole PTS reaction speed; **Location **is of the molecular subcellular localization regulation mechanism; **(P)HPr::GlgP **represent the process of the binding of (P)HPr::GlgP catalyzing glycogen decomposition. This is the snapshot of main part of our HFPN model, other components can be found in Additional files in URL [36].

The simulation of our HFPN models are conducted on Cell Illustrator 4.0 [35]. Before realizing a whole model, we have first set up two independent HFPN models based on these published ODE models of glycolysis and PP pathway [12,33] (see Additional file 1: Model-1 [36], HFPN models of this work (Mode-1 in Additional file 1, Mode-2 in Additional file 2, Mode-3 in Additional file 3 and Mode-4 in Additional file 4) and their simulation results (Figure S in Additional file 5), approaches (Methods in Additional file 6) and parameters (Table S in Additional file 7) are supplied in URL [36].) and mass balance theory models of PTS [13,34] (see Additional file 2: Model-2 [36]). Subsequently, these two HFPN models are combined into one (see Additional file 3: Model-3 [36]). This combined HFPN model was further extended by incorporating glycogen metabolism pathway and basic regulatory mechanisms, and finally we got an extended HFPN model of carbohydrate metabolism, as shown in Figure 2. We employed general mass action method to construct this integrated HFPN model (see Additional file 4: Model-4 [36]), in which mass action constants were manually fitted so as to meet biological data of glycogen and other metabolites concentrations from our former study [1]. For example from G6P to G1P to glycogen via ADPG, we obtained their mass action factors by adjusting the model's behavior to the biological data: that is, mass action parameters were determined based on the known information of concentrations of reactants and products. In order to check the availability of our integrated model (Model-4, see Additional file 4[36]), we made a comparison between the results from this model and from the model of glycolysis and PP pathway [12], showing the consistency of these two models (Figure S1, see Additional file 5[36]).

**Figure 3:**
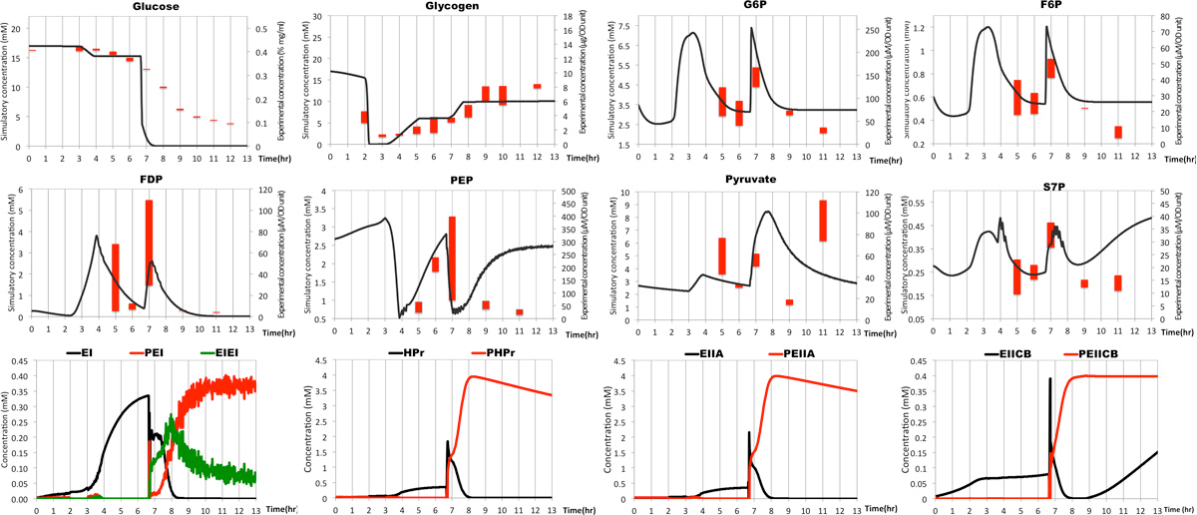
**Simulation results of HFPN model of extended center metabolism pathway in E. coli**. **Solid-curve **is simulation result of this work. **Red-bar **denotes experimental data of our previous work [1]. From these results, (1) when glucose is present, PTS enzymes are in a non-phosphorylated state; and when there is no glucose, they are phosphorylated; (2) the first peak of glycolysis and PP pathway metabolites occur just after glycogen is consumed; (3) the second peak of these metabolites is due to the glucose uptake. The whole simulation results are in Figure S5 (see Additional file 5).

The integrated HFPN model produced the correct behavior of metabolite concentrations of G6P, PEP, FDP etc. in a batch culture as well as the concentrations of glycogen and extracellular glucose in Figure 3, which can be confirmed by comparing with their experimental data in Supplementary data of [1]. Further, PTS enzymes level are also illustrated in Figure 3. In order to evaluating these simulation results, we calculated their Pearson product-moment correlation coefficient values (r-value) [37] against their experimental data (Figure S2 in Additional file 5[36]). Fortunately, r-valure shows simulation results of important metabolites for this study (G6P, F6P, FDP) are reliable (*|*r*|>*0.8). Those metabolites of *|*r*|<*0.8 should be addressed in the future, their low r-value may come from the lack of other routes connecting to PEP and PYR (for example, acetate pathway, gluconeogenesis etc.), or may come from the parameters of PTS part, which were conducted in vitro [13].

### Confirmation of the role of HPr and EIIA*^Glc ^*as key regulators by simulation

#### Biological analyses based on the simulation results

**Figure 4:**
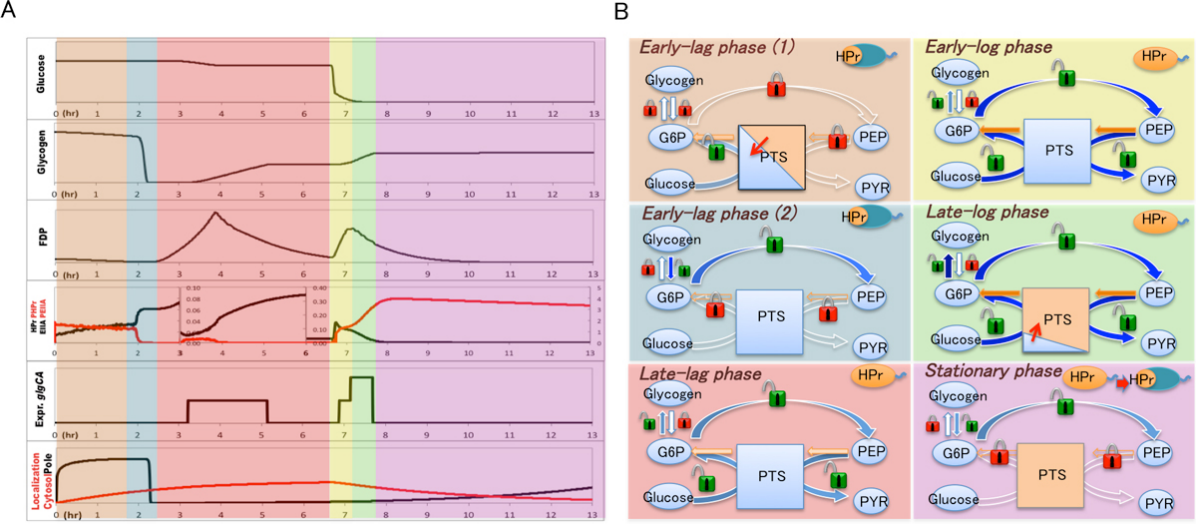
**Systematically understanding of the phases of extent center metabolism in an E. coli along its whole lifetime**. **A **illustrates experiment and simulation behaviors of some major metabolites and enzymes, **B **shows sugar and phosphate flux processes of *E. coli *utilizing glucose and glycogen. **Same color background areas of A and B are of the same phases**. In **B**: **Blue colored PTS **represents unphosphorylated PTS, **orange colored PTS **is phosphorylated PTS. **Blue filled arrows **indicate carbon flux routes, in which deeper blue color represents more flowing amount; **orange color filled arrows **indicate phosphate flux routes, in which deeper orange color represents more flowing amount. **Closed red locks **means inactivated pathway; **open green locks **means activated pathway. **The whole orange colored E. coli marked with "HPr" **indicates HPr is scattered in cytosol; **only orange colored pole E. coli marked with "HPr" **indicates HPr is at poles.

With running simulations on the constructed HFPN model, we are able to systematically understand the process of carbohydrate metabolism in a batch culture in *E. coli *along its lifetime, which consists of 5 phases, early lag phase, late lag phase, early log phase, late log phase, and stationary phase (Figure 4B). Simulated concentrations of glucose, glycogen, FDP, HPr (EIIA*^Glc^*), PHPr (PEIIA*^Glc^*), cAMP, *glgCA*, and (P)HPr subcellular localization are shown in Figure 4A.

***Early lag phase (1)***. At the beginning of this phase, *E. coli *begins its growth just after being put into a fresh medium. At this point, (P)HPr is mainly present at the poles and causes a little glucose uptake locally. Glycogen is not utilized well in this phase, because it is surrounded by PHPr. Indeed the higher affinity of PHPr than HPr isolates GlgP from glycogen, resulting in a very slow speed decomposition rate of glycogen.

***Early lag phase (2)***. Although this phase begins with PHPr, this protein slowly loses its phosphate. Because glycolytic pathway is not working in this phase, so phosphate cannot be provided through PTS. As HPr dephosphorylation completes, glycogen catalysis by HPr::GlgP begins, and *E. coli *uses glycogen as its main carbon source. Along with the quick consumption of glycogen, HPr is moved to the cytosol by the function of PEI [20]. Meanwhile, glycogen supplied phosphate flows into the central metabolic pathway, causing PEP accumulation. Distribution of (P)HPr in the cytosol will be finished at almost the same time.

***Late lag phase***. This is a period of slow glucose uptake, which is caused by a relevant lower level of PEP, due to a low speed EI dimerization [18]. This means that metabolites produced from glycogen support the transportation of phosphate for glucose uptake. During this period, (P)HPr has been distributed in the cytosol, whose major role is to work for PTS not for glycogen, and this also causes the start of glycogen accumulation. Meanwhile in this phase more PTS enzymes are expressed, preparing for the impending log phase.

***Early log phase***. Uptake of glucose is very fast in this phase due to the highly expressed PTS proteins and the active transportation of phosphate by these PTS proteins. Glucose is the main sugar source in this phase.

***Late log phase***. In this phase, under the combined regulation of PEIIA*^Glc ^*(via cAMP/CRP), and FDP (via Cra), *glgC *and *glgA *are expressed at extremely high levels [2,6,8], causing efficient glycogen accumulation. Due to the lower speed of phosphate output from the PTS comparing with its input speed from PEP, high level of PHPr are working for glucose uptake. (P)HPr is mainly expressed in the cytosol, so it can hardly contribute to glycogen decomposition.

***Stationary phase***. When cells come to a stationary phase, glycogen is in its slow speed catalyzing state. Since (P)HPr is maintained in phosphorylated state, it concentrates towards the poles, where glycogen is located. In the post stationary phase, there is no glucose supplied outside, glycogen is used as a carbon source for cells to survive. Glycogen low speed catalyzation is regulated by surrounding PHPr in poles. Next, if the *E. coli *is put into another culture, a new lag phase begins.

#### Qualitative description of regulator states throughout the phases

*Multi-valued formulation. glgC *and *glgA *are the genes that forms an operon with *glgP *[2,17,24]. According to experimental result, *glgC *and *glgA *are regulated by cAMP [2,17] and FDP [6], respectively. Hence we can consider that the transcriptions of these two genes are regulated by the combination of FDP amount and cAMP level, which are distinguished *α *(*glgC *&*glgA *activation) and *β *(*glgC *&*glgA *activation), respectively. Actually, from the biological literature [2,6,17], it is known that the composition speed of glycogen varies depending on the expression pattern of *α *and *β*. If either *α *or *β *is expressed, glycogen is composed in slow speed, but if both *α *and *β *are expressed, glycogen is composed in high speed. This function can be expressed by multi-valued formulation as presented in Table 1.

**Table 1:** Multi-valued formulation of the regulation in utilizing glycogen and glucose in *E. coli.*

*α* *glgC *&*glgA * activation	*β* *glgC *&*glgA * activation	*γ * glycogen composition
0	0	0
0	1	1
1	0	1
1	1	2

*α *represents *α *(*glgC *&*glgA *activation), *β *represents *β *(*glgC *&*glgA *activation), *γ *represents *γ *(glycogen composition). The meaning of multi-values are 0 (no/off), 1 (on/slow), and 2 (fast).

*Phase transitions based on the regulatory factors*. According to aforementioned analysis, the importance of HPr and EIIA*^Glc ^*on glycogen regulation is pointed out from a biological point of view. To make it more precise, we will express this regulatory system from an engineering point of view, presenting qualitative description of this system as shown in Table 2. Glycogen process is controlled by the regulators FDP, EIIA*^Glc^*, and HPr in the left column of this table. Among them, FDP and EIIA*^Glc ^*are involved in glycogen synthesis, and HPr in its decomposition. In the following, we will show, phase by phase, how composition and decomposition take place on the controls with these regulators in this table.

**Table 2:** Behaviors of key regulators (HPr and EIIA*^Glc^*) adjusting glucose and glycogen utilization in an *E. coli.*.

Regulator		Lag phase		Log phase		Stationary phase
		
		Early	Late	Early	Late	
**speed of glucose uptake**		very slow	slow	very fast	fast	no

**FDP amount**		low	high	low*→*high	high*→*low	no

*α***(glgC**&**glgA activation)**		off	on	off*→*on	on*→*off	off

**EIIA***^Glc ^* **phosphorylation**		yes*→*no	no	no	yes	yes

**(regulated cAMP level)**		high*→*low	low	low	high	high

*β***(glgC**&**glgA activation)**		on*→*off	off	off	on	on

**glycogen**	*γ***(composition)**	no	slow	slow	fast*→*slow	no

	**(decomposition)**	slow*→*fast	no	no	no	slow

**HPr**	**(phosphorylation)**	yes*→*no	no	no	yes	yes

	**(localization)**	pole	cytosol	cytosol	cytosol	pole

In this table, the five proliferation phases (e.g. Late lag phase) are corresponding with their processes of experiment and simulation data in Figure 4.

***Early lag phase***. Because of "very slow" uptake speed of glucose, FDP amount is in "low" level, resulting in "off" expression of *glgC *&*glgA *genes (*α*). EIIA*^Glc ^*and HPr display the same behavior, changing these phosphorylation states, "yes *→ *no". In addition, *glgC *&*glgA *activation (*β*) is influenced by this state transition as "on *→ *off" in Table 2. Glycogen composition, however, is not influenced by these regulations, because the uptake speed of glucose is too slow to produce glycogen. On the other hand, glycogen decomposition takes place in this phase, with changing its speed "slow *→ *fast" according to the phosphorylation state transition of HPr "yes *→ *no". Hence, glycogen is the major sugar source in this phase.

***Late lag phase***. Since *E. coli *has not consumed much energy yet in this phase, FDP accumulates in "high" levels despite the "slow" glucose uptake speed. Hence *glgC *&*glgA *(*α*) is "on". On the contrary, *glgC *&*glgA *(*β*) is "off", which is resulted from "no" phosphorylation state of EIIA*^Glc ^*via "low" cAMP level. According to the rule (if *α *= 1 and *β *= 0 then *γ *= 1) in Table 1, glycogen is composed (*γ*) in "slow" speed. On the other hand, glycogen decomposition does not take place in this phase, because HPr is not located at the poles but distributed in the cytosol, which does not satisfy the requirement for glycogen decomposition.

***Early log phase***. Due to "very fast" speed of glucose uptake, FDP is accumulated in *E. coli*, despite its high metabolic activity, changing its amount as "low *→ *high". Accordingly, the state of *glgC *&*glgA *(*α*) activation is changed as "off *→ *on". In this stage, HPr is not phosphorylated, then the expression of *glgC *&*glgA*(*β*) is "off"; consequently the composition speed of glycogen (*γ*) is "slow", though it temporally drops to "no" level. On the other hand, "no" decomposition of glucose takes place in this phase from the same reason as late lag phase above.

***Late log phase***. Because much glucose was consumed in the previous phase, its uptake speed is going to be slow down. Accordingly, for the phosphate flow in PTS, the input speed of phosphate from PEP becomes faster than the output speed to G6P, causing EIIA*Glc *phosphorylation "yes" and cAMP level "high". As a result, *glgC *&*glgA *activation (*β*) turns "on". In addition, because, in the early half of this phase, FDP is in a high level, *glgC *&*glgA *activation (*α*) is also turned "on". Hence, both *α *and *β *regulations are working. In this case, according to Table 1, glycogen composition (*γ*) should be marked at "fast" speed. Accompanying with decreasing glucose amount, FDP concentration drops later in this phase, that is "high *→ *low", resuling in *glgC *&*glgA *activation (*α*) as "on *→ *off". As a result, in the later part of this phase, the speed of glycogen composition (*γ*) changes as "fast *→ *slow", because degragation of enzyme needs time. On the other hand, in this phase, HPr is still in cytosol working for PTS, not for glycogenolysis. In all, since "fast" composition and "no" decomposition are conducted, glycogen accumulates quickly in this period.

***Stationary phase***. In this period, because extracellular glucose has been totally consumed off, the speed of glycogen is marked as "no" despite the "on" state of *glgC *&*glgA *activation (*β*). Hence there is "no" glycogen composition (*γ*). Because of the inactive PTS and the high amount glycogen, (P)HPr is concentrated at the "poles", decomposing glycogen (*γ*) in a "slow speed" for long survival of cells.

## Conclusion

Some works focus on modeling glycolysis, pentose phosphate pathway, TCA cycle etc. [12,26,27], and some focus on the calculation of PTS performance with a protein mass balance theory method [13,34]. And also some of them set up ODE models by combining PTS into their glycolysis pathways [26,27]. But none of them take the glycogen metabolic pathway into account. In this work we firstly integrated general mass action based glycogen metabolism model, mass balance theory based PTS model, and ODE model of glycolysis and PP pathway into a computational model with HFPN.

By applying this model, basic regulators for *E. coli *to utilize extracellular glucose and intracellular glycogen were identified. That is, (P)HPr not only works as a member of PTS enzymes but also functions to realize different catalyzing speeds of glycogen by its phosphorylation state combined with GlgP. Actually, phosphorylation state of (P)HPr is controlled by the phosphate flux speed influx and outflux of PTS, and this flux speed is controlled by gene expression, subcellular localization, and metabolite concentration (glucose, PEP, FDP). HPr and EIIA*^Glc ^*are considered to be key roles among these regulators during the utilization of glycogen and glucose by *E. coli*.

Based on the model with regulatory systems in this work, we provided a systematic view of glucose and glycogen utilization by *E. coli*. This confirms our previous conclusion that glycogen plays an important role as a primary carbon source in lag phase [1].

## Methods

Before achieved our final version integrated HFPN model (Model-4, see Additional file 4[36]), which shows the dynamic time course model of extended central metabolism pathway (glycolysis, pentose phosphate (PP) pathway, glycogen metabolic pathway, PTS and regulators), we have set up 3 preliminary models: a model of glycolysis and PP pathways (Model-1, see Additional file 1[36]), transplanted from ODE models; a model of PTS (Model-2, see Additional file 2[36]), according to mass balance theory based PTS models; and a combined model of glycolysis, PP pathway and PTS (Model-3, see Additional file 3[36]). By applying regulatory systems and glycogen metabolism network to Model-3, a dynamic HFPN model of central metabolism is settled as Model-4. The method of biological experiment was explained in our previous paper [1], of which the current study is the continuation. More detail modeling approach is in Additional file 6[36].

## List of abbreviation used

Hybrid functional Petri net (HFPN), ordinary differential equation (ODE), pentose phosphate pathway (PP pathway), metabolite level (M-level), molecule localization level (L-level), phosphate flux level (F-level), protein level(P-level), gene expression level (G-level), phosphorylated enzyme E (PE) (e.g. PHPr), chemical Binding (A::B) (e.g. HPr::GlgP), phosphoenolpyruvate:sugar phosphotransferase system (PTS) glucose 6-phosphate (G6P), glucose 1-phosphate (G1P), ADP-glucose (ADPG), fructose 6-phosphate (F6P), fructose-1,6-bisphosphate (FDP), phosphoenolpyruvate (PEP), pyruvate (PYR),D-glyceraldehyde 3-phosphate (GAP), dihydroxyacetone phosphate (DHAP), 1,3-bisphospho-D-glycerate (PGP), 3-phospho-D-glycerate (3PG), 2-phospho-D-glycerate (2PG), 6-Phosphogluconolactone (6PG), ribulose 5-phos- phate (Ribu5P), ribulose 5-phosphate (R5P), xylulose 5-phosphate (Xyl5P), erythrose 4-phosphate (E4P), sedoheptulose-7-phosphate (S7P), *ptsHIcrr *(PTS enzymes operon), *glgBXCAP *(glycogen enzymes operon).

## Competing interests

The authors declare that they have no competing interests.

## Authors' contributions

Z. Tian performed the study of modeling and data analysis. A. Fauré and H. Matsuno worked on this study with giving comments to Z. Tian. H. Mori worked on biology knowledge support. All authors approved the final manuscript.

## Supplementary Material

Additional file 1 **Model-1**. Cell Illustrator 4.0 file of HFPN model of central metabolism pathway (glycolysis and pentose phosphate pathway), which can be download from URL [36]. Click here for file

Additional file 2 **Model-2**. Cell Illustrator 4.0 file of HFPN model of PTS, which can be download from URL [36]. Click here for file

Additional file 3 **Model-3**. Cell Illustrator 4.0 file of HFPN model of combination of central metabolism pathway and PTS, which can be download from URL [36]. Click here for file

Additional file 4 **Model-4**. Cell Illustrator 4.0 file of dynamic HFPN model of central metabolism pathway, PTS and glycogen metabolism with the regulatory mechanisms, which can be download from URL [36]. Click here for file

Additional file 5 **Figure S**. Additional Figures, which also can be download from URL [36]. Click here for file

Additional file 6 **Methods**. Detail Methods, which also can be download from URL [36]. Click here for file

Additional file 7 **Table S**. Additional Tables, which also can be download from URL [36]. Click here for file

## Declarations

Publication of this article was funded by Grant-in-Aid for Scientific Research on Innovative Areas "Synthetic Biology" (24119509) from JSPS.

This article has been published as part of *BMC Systems Biology *Volume 7 Supplement 6, 2013: Selected articles from the 24th International Conference on Genome Informatics (GIW2013). The full contents of the supplement are available online at http://www.biomedcentral.com/bmcsystbiol/supplements/7/S6.
