# Supplementary material for: Identification of key regulators in glycogen utilization in E. coli based on the simulations from a hybrid functional Petri net model
Source: BMC Syst Biol. 2013 Dec 13;7(Suppl 6):S1. doi: 10.1186/1752-0509-7-S6-S1 (PMC4029488; doi:10.1186/1752-0509-7-S6-S1)
Supplement: Additional file 5 — Figure S. Additional Figures, which also can be download from URL [36]. [file 1752-0509-7-S6-S1-S5.PDF]

Additional Figures (Figure S) of [Z. Tian et al (2013)]

Figure S1 Comparison of our integrated model (Model-4) and Chassagnole's model of [Chassagnole2002]. 2

Figure S2 R-values of Experiment data of [1] and simulation results of Model-4. .. 3

Figure S3 Model-1: An ODE based HFPN model of central metabolism pathway.. 4

Figure S4 Model-2: A mass balance theory based HFPN model of PTS. 5

Figure S5 Model-3: A combined HFPN model of Model-1 & Model-2. 6

Figure S6 Simulation results of Model-4. 7

Figure S7 Control boxes of Model-4. 8

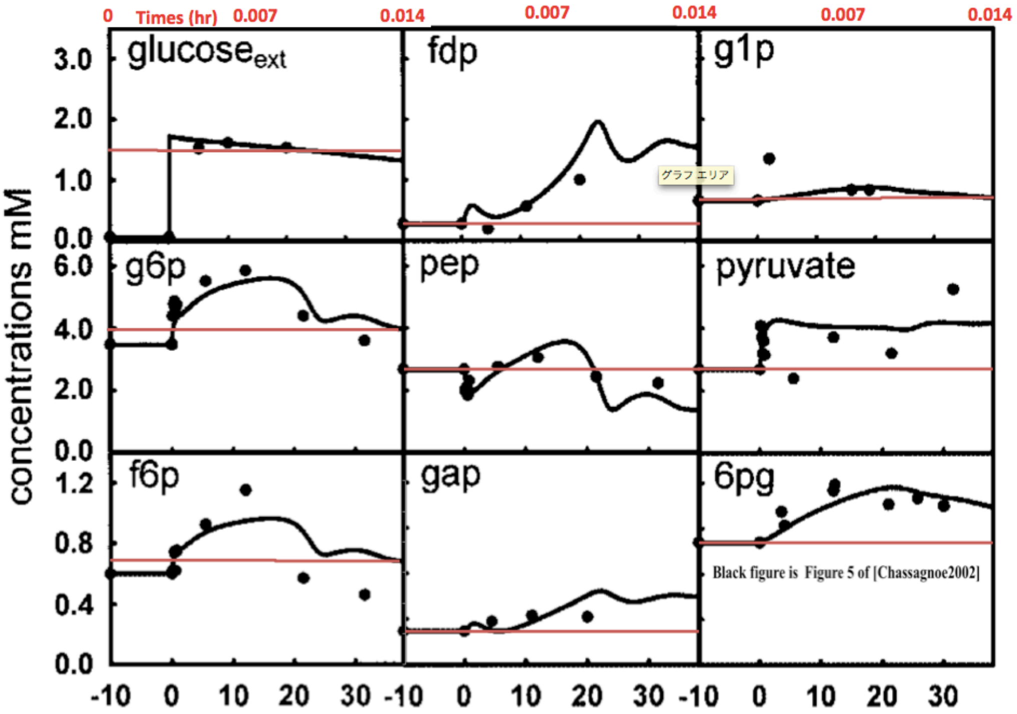

Figure S1 Comparison of our integrated model (Model-4) and Chassagnole's model of [Chassagnole2002].

Red color curves are the simulation results integrated model of this work (Model-4), whose initial value of glucose is 1.5 mM and simulation time length is 0.14 hr (≈50 s). **Black solid lines and points** are simulation results and experimental data of [Chassagnole2002] repetitively.

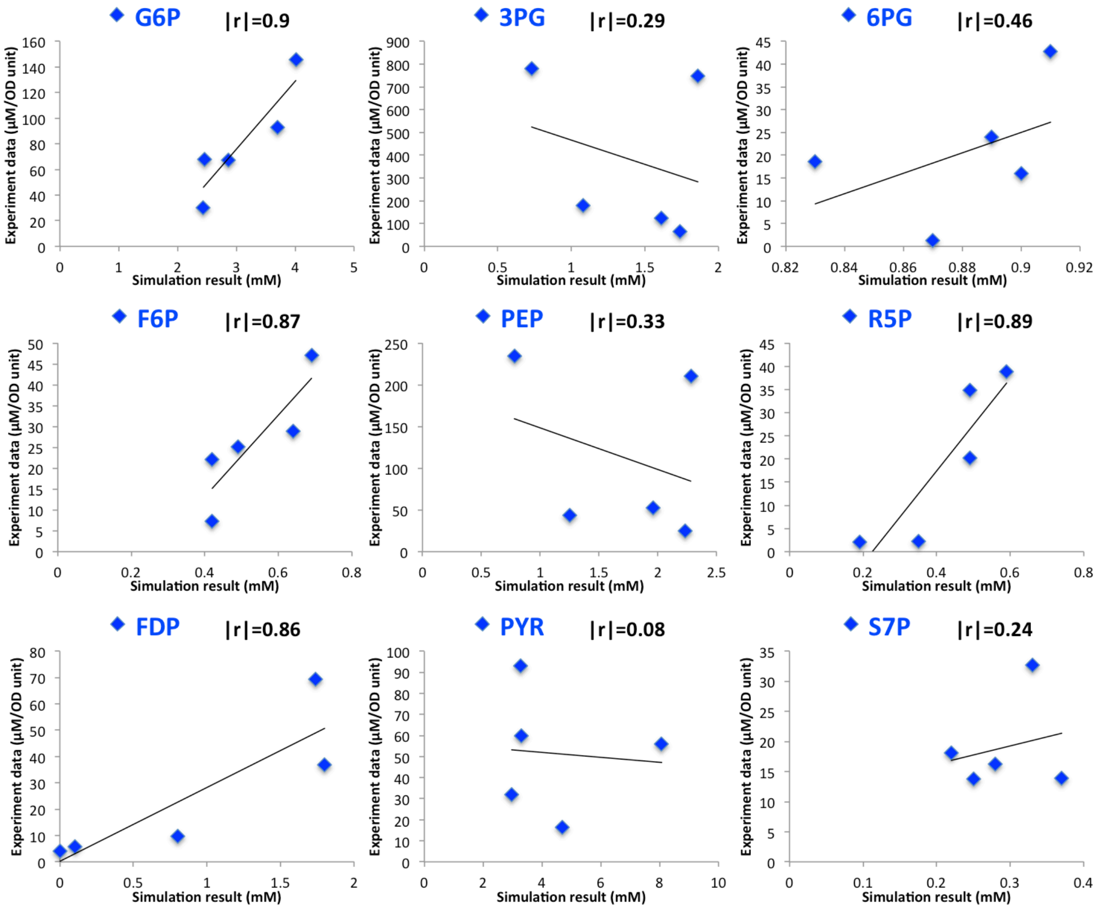

Figure S2 R-values of Experiment data of [1] and simulation results of Model-4. **|r|** is Pearson product-moment correlation coefficient value (r-value). **Horizontal axis** denotes simulation results of this study (Model-4). **Vertical axis** are experimental data from our previous study [1].

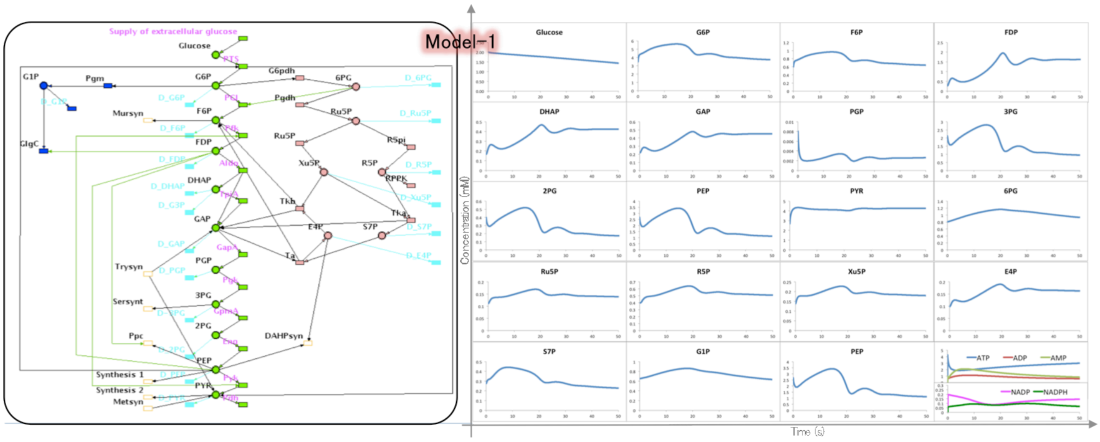

Figure S3 Model-1: An ODE based HFPN model of central metabolism pathway. **Left panel** is snapshot of Model-1 (HFPN model of Cell Illustrator 4.0 is in **Additional file 1: Mode-l**), including glycolysis (green filled components), PP pathway(pink filled components), and a part of glycogen metabolism pathway (blue filled components). Meanwhile, cyan colored transitions are delusion of it upstream metabolites, and oranged boarder transitions are linking to to other kinds of pathways. **Right panel** is the simulation results of Model-1, in which each result is same to its original ODE model result in Figure 2 of (Chassagnole et al. 2002). Total simulation time course is 50 petri net time (pt) in Model-1, equals to 50 s in ODE model of (Chassagnole et al. 2002).

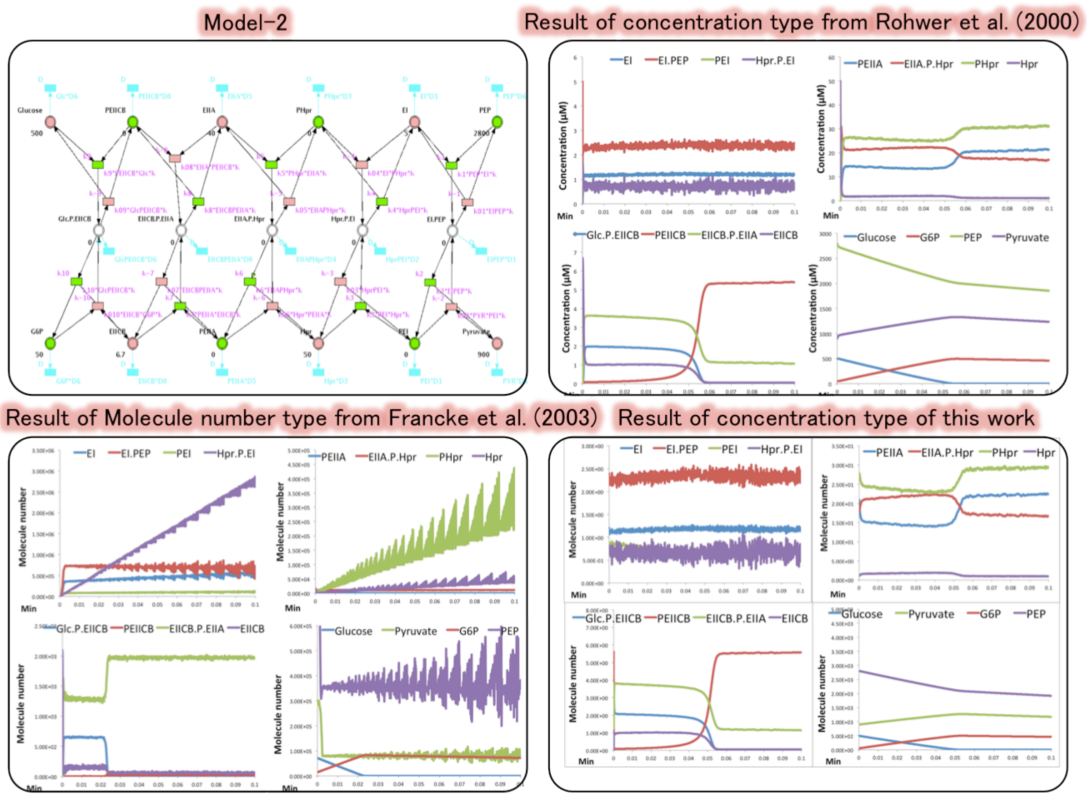

Figure S4 Model-2: A mass balance theory based HFPN model of PTS.

Upper left panel is snapshot of Model-2. **Upper right panel** is simulation results of **concentration** type Model-2, whose parameters are from (Rodríguez et al. 2006; Rohwer et al. 2000) (HFPN model of Cell Illustrator 4.0 is in **Additional file 8: Mode-2 (Conc.)**). **Lower left panel** is simulation results of **molecule number** type Model-2, whose parameters are from (Francke et al. 2003) (HFPN model of Cell Illustrator 4.0 is in **Additional file 9: Mode-2 (Mole.)**). **Lower right panel** is simulation results of concentration type Model-2, whose initial values are defined by and used in this work (HFPN model of Cell Illustrator 4.0 is in **Additional file 2: Mode-2**). These initial values list in Table S4.

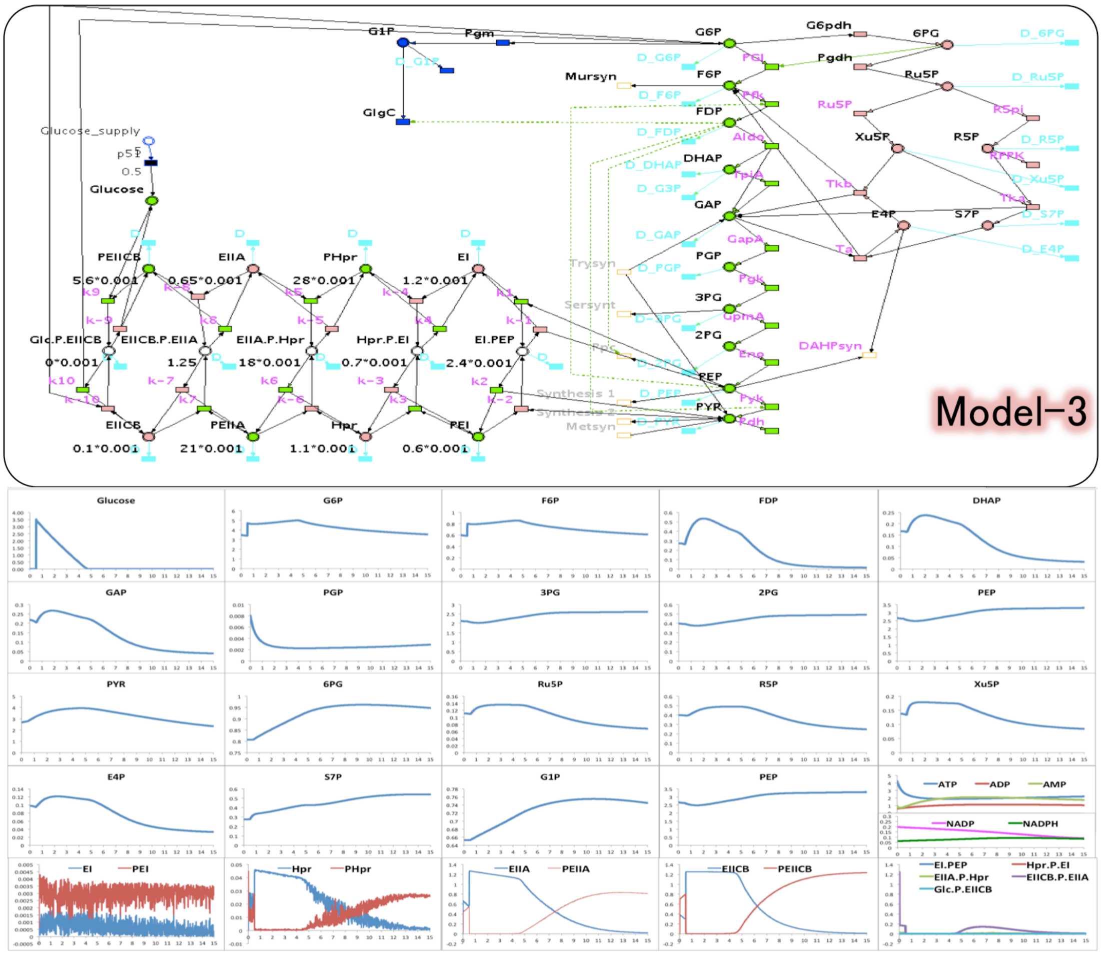

Figure S5 Model-3: A combined HFPN model of Model-1 & Model-2.

Upper panel is snapshot of Model-3. **Lower panel** is simulation results of Model-3, (HFPN model of Cell Illustrator 4.0 is in **Additional file 3: Mode-3**).

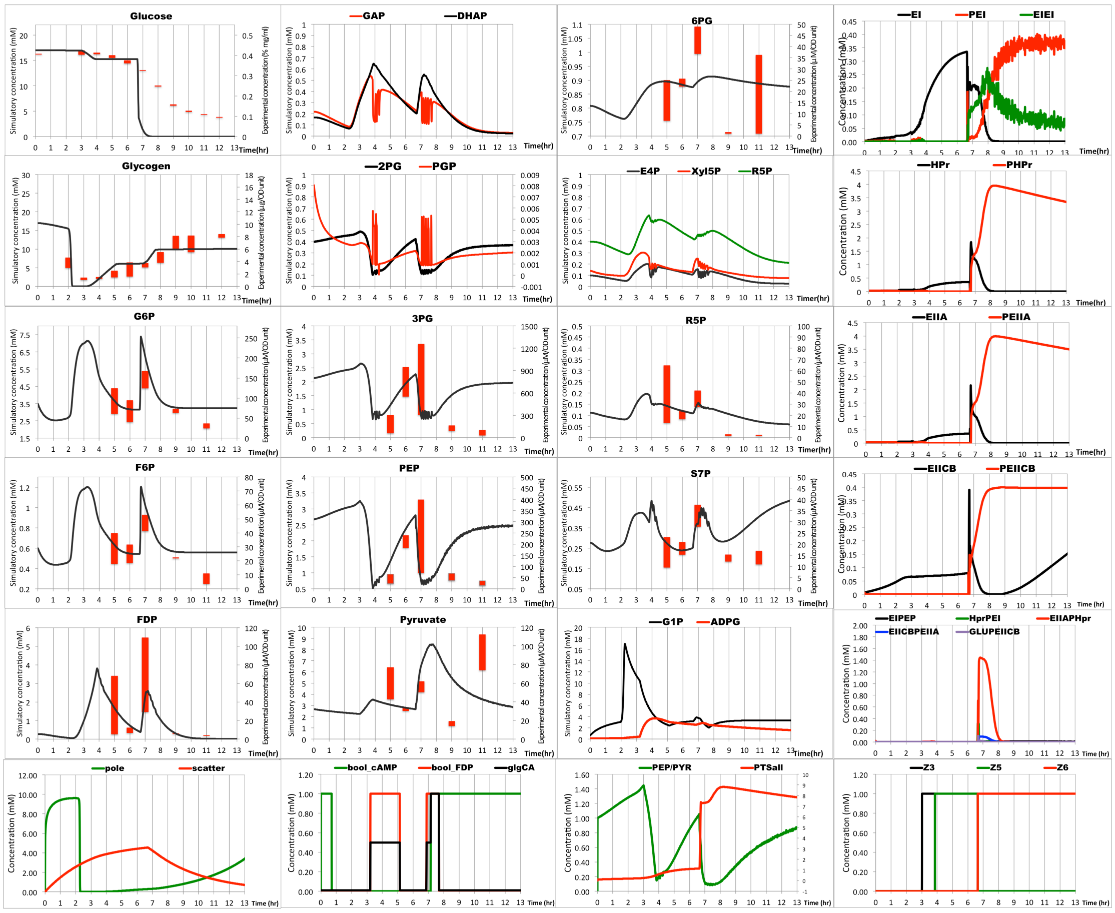

Figure S6 Simulation results of Model-4.

(HFPN model of Cell Illustrator 4.0 is in **Additional file 4: Mode-4**) **Solid curve** is computational result of this work. **Bar** denotes experimental data from our previous study [1]. The 4 bottom patterns are of the regulation mechanism, **Bottom left 1st pattern**: HPr localization in cytosol (curve "scatter") or at poles (curve "pole"); **Bottom left 2nd pattern**: gene expression of *glgC* and *glgA* (curve "*glgCA*") is controlled by cAMP (curve "bool cAMP") and FDP (curve "bool FDP"), from left to right. **Bottom right 1st and 2nd patterns**: Different phosphate transportation speeds in the whole or parts of PTS (curve "Z3", "Z5", "Z6") are controlled by PTS genes expression level (curve "PTSall") and PEP to Pyruvate ratio (curve "PEP/PYR ratio").

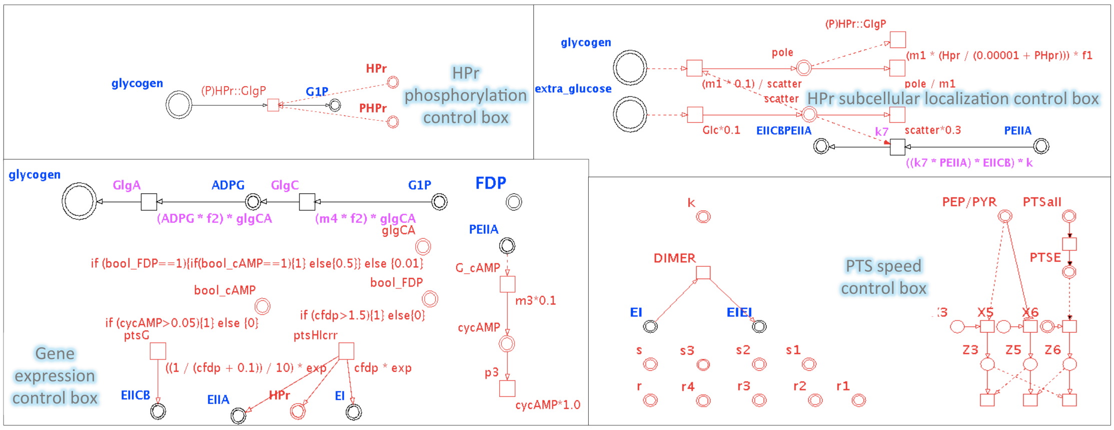

Figure S7 Control boxes of Model-4.

Upper left panel: HPr phosphorylation control box **Upper right panel:** HPr subcellular localization control box. **Lower left panel:** gene expression control box. **Lower right panel:** PTS speed control box.
