# Supplementary material for: Identification of key regulators in glycogen utilization in E. coli based on the simulations from a hybrid functional Petri net model
Source: BMC Syst Biol. 2013 Dec 13;7(Suppl 6):S1. doi: 10.1186/1752-0509-7-S6-S1 (PMC4029488; doi:10.1186/1752-0509-7-S6-S1)
Supplement: Additional file 6 — Methods. Detail Methods, which also can be download from URL [36]. [file 1752-0509-7-S6-S1-S6.PDF]

Methods

Before achieved our final version integrated HFPN model (Model-4, see AddFile 4 [36]), which shows the dynamic time course model of extended central metabolism pathway (glycolysis, pentose phosphate (PP) pathway, glycogen metabolic pathway, PTS and regulators), we have set up 3 preliminary models: a model of glycolysis and PP pathways (Model-1, see AddFile 1 [36]), transplanted from ODE models; a model of PTS (Model-2, see AddFile 2 [36]), according to mass balance theory based PTS models; and a combined model of glycolysis, PP pathway and PTS (Model-3, see AddFile 3 [36]). By applying regulatory systems and glycogen metabolism network to Model-3, a dynamic HFPN model of central metabolism is settled as Model-4. The method of biological experiment was explained in our previous paper [1], of which the current study is the continuation. More detail modeling approach is in AddFile 6 [36].

Model-1: An ODE based HFPN model of glycolysis and PP pathway

In this work, HFPN model of glycolysis and PP pathway were borrowed from Chassagnole et al. (2002) ODE model [12], which has been well stored in database BioModels [33]. We imported this model to Cell Illustrator 4.0 [35], with a little modification for technically keeping a consistency in time scale. As a consequence, a HFPN model of glycolysis and PP pathways was established (see AddFile 1). Our simulation results (Figure S3(see AddFile 5) shows the same behaviors of metabolites (glucose, G6P, PEP, etc.) and cofactors (ATP, ADP, etc.) with that in Figure 2 of [12] , which indicates an ODE model could be transplanted into a HFPN model seamlessly (Equations and parameters list in Table S1 and Table S2 of AddFile 7).

Model-2: A mass balance theory based HFPN model of PTS

Rohwer et al (2000) [13]. founded a mass balance theory based model of PTS in steady state, by using experimentally tested mass action constant for each elementary biochemical reaction within PTS enzymes, which leads many subsequent studies [9,34]. Manually setup mass balance theory based HFPN model of PTS and taking mass action constants from [9, 34], we set up two types of PTS models in Cell Illustrator 4.0 [35]: one type, initial values of enzymes are are μM (concentration) (see AddFile 2), another type, the initial values are molecules number. By comparing the upper panel with the lower panel of Figure S4 (see AddFile 5), we could found results of μM (concentration) type of model performs better than the molecular number type one, which is because of high value causes data overflow.

Mass balance theory based models of [13, 34] run in time course of seconds (e.g. $k_1 = 32.7\ \mu M^{-1}s^{-1}$), of Francke et al. (2003) [9] is time course of minutes (e.g. $k_1 = 1960\ \mu M^{-1}min^{-1}$). We tested both kinds of time courses, the μM (concentration) type Model-2 (see AddFile 2) works well (Equations and parameters are in Table S3 and S4 (see AddFile 7)), and its simulation results in Figure S4 (see AddFile 5) show similar tendency with those of [34] .

On the other hand, from our simulation results in Figure S4 (see AddFile 5), we could see: When there is glucose supplied, most of unphosphorylated PTS enzymes (e.g. EIICB) and Combination compounds (e.g. EIICB::P::EIIA) are in higher concentrations. When there is no glucose supplied, most of phosphorylate PTS enzymes

(e.g. PEIICB) are in higher concentrations; these results support the statement in [8].

In the HFPN Model-2 of PTS (see AddFile 2), glucose is consumed off at 0.05 pt (petri net simulation time), that is after this time, model comes to its steady state without glucose. Since in biological experiment, an E. coli is often taken in to a glucose rich culture from stationary phase, accordingly we reseted the initial values of PTS enzymes (Table S4 in AddFile 5), and adjusted Model-2 Additional file, equations and parameters are Table S3 and S5 in AddFile 7)), and we could find its availability from its calculation results (Figure S4 lower right panel in AddFile 5).

Model-3: A combined HFPN model of Model-1 & Model-2

Attempt to combining Model-2 (Mass balance theory based model of PTS of AddFile 2) with Model-1 (ODE based central metabolism pathway model of AddFile 1), our first step is normalizing the parameters of the two different types of HFPN models. Since components concentration unit of glycolysis and PP pathway model is mM and PTS model is μM, we decreased the values of PTS enzymes concentration 1000 times. Boundary metabolites concentrations (Glucose, G6P, PEP and Pyruvate) are different in these two models, Pyruvate is 900 μM in Model-2 (see AddFile 2) and 2.67 mM in Model-1 (see AddFile 1) . Via behavior testing, we found initial values of boundary metabolites in Model-1 (see AddFile 1) are better.

From the time course aspect, Model-1 (see AddFile 1) is in 50 s, as while, Model-2 (see AddFile 2) is in 0.1 min. Both these two time lengths are too short for our purpose, which is to simulate whole process of *E. coli* utilizing glucose and glycogen. On the other hand, high reaction speed needs more detail calculation fineness to get beautiful results, but too detail calculation fineness requires plenty calculating time in Cell Illustrator 4.0. Thus we have to balance the efficiency and calculation fineness in this combined HFPN model.

Deleting PTS process and all its connected arcs of Model-1 part (see AddFile 1), Deleting boundary metabolites (glucose, G6P PEP and Pyruvate entities) of Model-2 part (see AddFile 2), connecting Model-1 and Model-2 by boundary metabolites and associated processes. At last we omitted glucose supply method of Model-1, and assembled a sudden glucose supply in 0.5 pt in order to watch PTS and glycolysis and PP pathway sensitivity (Model-3 of AddFile 3). Fortunately, we found by simply control glucose supply way, we could extent our model to a long time range (hours). This achievement may somehow prove the robustness of the parameters and equation of the original experiments [12, 13].

Simulation results of Model-3 (shown in Figure S5 of AddFile 5) could be understood as: When there is glucose supply, most PTS enzymes are in unphosphate state (HPr. EIIA, EIICB), and while there is no glucose, they are in phosphate state (PHPr. PEIIA, PEIICB), and these behaviors coordinate with the conclusion of [7]. Further, metabolites concentrations waved when glucose supply status changes, this could be a proof of the sensitivity of glycolysis part against glucose supply from PTS part. Equations and parameters in Model-3 (see AddFile 3) are same to corespondent parts in Model-1 (see AddFile 1) and Model-2 (see AddFile 2), despite the only difference: place "glucose" initial value is 0, while it is supplied 5 unit in one time by "glucose_supply" at 0.5 pt (see Figure S5 of AddFile 5).

Model-4: A dynamic HFPN model of combination of Model-3, regulation mechanisms, and glycogen metabolism pathway

Via adding a glycogen metabolism pathway in Model-3 (see AddFile 3), a HFPN model backbone of extended carbohydrate metabolism pathway is constructed (Model-4 of AddFile 4). Though Model-1 (see AddFile 1) includes a part of glycogen metabolism pathway (include: G1P, PGM, and GlgC (see Figure S3 in AddFile 5)), its designed PGM transition is in mono-direction, which can not realized the bi-direction function. We applied mass action method to construct glycogen metabolism pathway, whose mass action constants were manually fitted by meeting the biological data of glycogen and other metabolites concentrations from our former study [1], together with regulatory mechanisms. In our integrated HFPN model (Model-4, see AddFile 4), the parts of glycolysis and PTS were inherited from Chasagnole's model (Model-1, see AddFile 1) [12] and Rohwer's model (Model-2, see AddFile 2) [13]. For those reactions lacking kinetic parameters, for example from G6P to G1P to glycogen via ADPG, we obtained their mass action factors by adjusting the model's behavior to the biological data: that is, mass action parameters were determined based on the known information of concentrations of reactants and products.

From a systematic point of view, our current model (Model-4, see AddFile 4) was designed to simulate the whole life metabolic process of *E. coli.* Consequently, the time scale of our model is in hours, whereas that of Chassagnole's model [12] is in seconds. Due to the difference in time resolution, direct comparison is difficult, as the whole 50 s simulated by Chassagnole [12],which's time length is almost as a moment in our simulation. To compare our results, we simulated our model starting from an initial glucose concentration of 1.5 mM, similar to that used by Chassagnole et al. [12]. The comparison is shown Figure S1 (see AddFile 5). Although our model cannot capture as much detail due to the smaller resolution, our simulation results are in the same range of magnitude as experimental data.

Simulation results of Model-4 (see AddFile 4) are showing in Figure S6 (in AddFile 5), in which concentration of glycolysis metabolites, glucose and glycogen also including experimental data of our former study [1]. Computational behaviors of PTS enzymes and regulators can be found in this figure also. We selected some typical results showing in our manuscript (Figure 4). And we introduced Pearson product-moment correlation coefficient value (r-value) to evaluate these simulation results (Figure S2 in AddFile 5), in order to check these patterns evolutionary tendencies (explained in last part of this chapter). Parameters, and threshold of arcs are listed in Table S6, S7 and S8 (see AddFile 7).

The basic regulatory mechanisms we elected in this work (see Figure 1 and 2), were realized by 4 control boxes in Model-4 (see AddFile 4), they are HPr phosphorylation control box, HPr subcellular localization control box, gene expression control box, and PTS speed control box. We isolated them in Figure S7 (see AddFile 5) from colors filled areas of Figure 3.

HPr phosphorylation control box

Different phosphorylation status' HPr binding with GlgP catalyze different glycogen decomposition speed [14], accordingly our glycogen phosphorylation transition

"(P)HPr::GlgP" is defined as:

$$glg * (Hpr/(0.00001 + PHpr)) * f1, \qquad (1)$$

in which, glg, Hpr, and PHpr are concentrations of glycogen, HPr, or PHPr respectively. f1 is a manual fitting parameter, 0.00001 is a guarantee for correctness of division calculation. This control box is illustrated in upper left panel of Figure S7 (see AddFile 5).

HPr subcellular localization control box
Lopian et al. (2010) made a detail study on subcellular location of PTS enzymes, PHPr movement from poles to cytosol is driven by PEI tranfering phosohate to it [20]. At first we tried to realized this control by only applying PEI to stimulate HPr translocation, but failed. This may because this is a more complex process in vivo than our thinking. Then we changed our mind to a systematic way, we use the two sugar supplies as the stimulation condition of HPr localization. This control are shown in upper right panel of Figure S6 (see AddFile 5) and follow these rules: (I) When glycogen is accumulated and glucose uptake stops, (P)Hpr will concentrate to poles around glycogen and work for it. (II) More glucose uptake, more (P)HPr scatter to cytosol, and wider gate of phosphate transportation from HPr to EIIA. (III) When (P)HPr locates in poles, it works for glycogen, when (P)Hpr is scatter in cytosol, it mainly work as a member of PTS.

In this control box, place "pole" controls transition (P)HPr::GlgP, while place "scatter" controls transition k7 of PTS. Since there are many transitions from HPr to EIICB (k6, k7, etc.), which one should be the controlling target of the cytosol scattered HPr? We tried to test them in Model-4 (see AddFile 4), at last we found the calculation results of PTS of k7 can meet biological theory of [8]. We predict HPr localization controls the phosphate transfer from PEIIA to EIICB (transition k7).

Gene expression control box
PEIIA enhances the production of global regulator cAMP, which together with CRP construct cAMP/CRP complex to control expression of glgC and glgA [17,38]. Global regulator Cra is inhibited by FDP and F1P [6], and it also regulates genes expression of either *glgCAP* and *ptsHIcrr* by directly binding to them, or indirectly via small RNA SgrS and SgrT pathway to *ptsG* [6]. At last we apply each of these gene express places in to their associated transitions. We use a logical control box in HFPN to realize this combined function (lower left panel of Figure S1 in AddFile 5). Try to achieve best control affect, we simplified their regulation pathways. *ptsG* and *ptsHIcrr* are directly received controlling signals from FDP, and *glgC* and *glgA* get signal from Boolean entities bool_cAMP and bool_FDP, which are abstracted from cAMP and FDP concentration.

PTS speed control box
This control box includes controls on EI dimerization speed and whole PTS reaction speed, in which PEP stimulates EI dimerization is the most difficulty part for us.

During our simulation, we adopted PEP/Pyruvate ratio to control the speed limitation of PTS [18], EI dimerization, could not meet both the biological requirements of metabolites concentration from [1] and the theory analysis of PTS behavior [8]. In this part we assumed: (I) When PEP/Pyruvate ratio reaches a certain high threshold, the "gate" of EI dimerization is opened, no matter this ratio is lower or higher than the threshold later. (II) When PTS enzymes expressed to a certain high level (threshold), the whole PTS reactions speeds will climb to an extremely high level.

We integrated a control matrix (lower right part of Figure S1 in AddFile 5), which includes three logical switch conditions, Z3, Z5, and Z6, which in charge of the speed changes: Z3, in middle lag phase, after PEP/PYR ratio gets to a threshold value because of the consumption of glycogen, a certain speed flux gate from EI to EIEI is turned on. Z5 is control by PEP/PYR ratio. Z6 control the speed from lag phase to log phase, which is generated by *pts* genes expression. Assembling these mechanisms in k, the whole PTS reaction speed coefficient, and in "EI_dimerize" transition, we got:

$$k = if(Z6 == 1)\{if(Glc > 4)\{s2\}else\{s3\}\}else\{s1\}, \tag{2}$$

$$\begin{aligned} DIMER = \; & if(Z6 == 1)\{EI * cpep * r6\} \\ & elseif(Z5 == 1)\{EI * cpep * r5\} \\ & elseif(Z3 == 1)\{EI * cpep * r3\} \\ & else\{EI * cpep * r0\}, \end{aligned} \tag{3}$$

in which, s2, s3, s1 are three speed coefficients of whole PTS, and r0, r3, r5, and r6 are three speed coefficients of EI dimerize.
