# Supplementary material for: Identification of key regulators in glycogen utilization in E. coli based on the simulations from a hybrid functional Petri net model
Source: BMC Syst Biol. 2013 Dec 13;7(Suppl 6):S1. doi: 10.1186/1752-0509-7-S6-S1 (PMC4029488; doi:10.1186/1752-0509-7-S6-S1)
Supplement: Additional file 7 — Table S. Additional Tables, which also can be download from URL [36]. [file 1752-0509-7-S6-S1-S7.PDF]

Additional Tables (Table S) of [Z. Tian et al (2013)]

Table S1 Transitions (Kinetic equations) of Model-1 2

Table S2 Places (Initial parameters or equations) of Model-1 4

Table S3 Equations of Model-2. 6

Table S4 Different sources initial values of PTS enzymes of Model-2. 7

Table S5 Parameters of Model-2 8

Table S6 Equations of Model-4. 9

Table S7 Parameters of Model-4 12

Table S8 Arcs' firing value of Model-4. 16

Table S1 Transitions (Kinetic equations) of Model-1

Transition	Kinetic Script	Source
Culture glucose	(extracellular * vEXTER_Dil *(vEXTER_cfeed - cglcex))	[12,28]
PTS	((extracellular * vPTS_rmaxPTS * cglcex *(cpep / cpyr))/((vPTS_KPTSa1 +(vPTS_KPTSa2 *(cpep / cpyr))+(vPTS_KPTSa3 * cglcex)+(cglcex *(cpep / cpyr)))*(1 +(pow(cg6p , vPTS_nPTSg6p)/ vPTS_KPTSg6p))))	[12,28]
PGI	((cytosol * vPGI_rmaxPGI *(cg6p -(cf6p / vPGI_KPGIeq)))/((vPGI_KPGIg6p *(1 +(cf6p /(vPGI_KPGIf6p *(1 +(cpg / vPGI_KPGIf6ppginh))))+(cpg / vPGI_KPGIg6ppginh)))+ cg6p))	[12,28]
Pfk	((cytosol * vPFK_rmaxPFK * catp * cf6p)/((catp +(vPFK_KPFKatps *(1 +(cadp / vPFK_KPFKadpc))))*(cf6p +((vPFK_KPFKf6ps *(1 +(cpep / vPFK_KPFKpep)+(cadp / vPFK_KPFKadpb)+(camp / vPFK_KPFKampb)))/(1 +(cadp / vPFK_KPFKadpa)+(camp / vPFK_KPFKampa))))*(1 +(vPFK_LPFK /pow((1 +((cf6p *(1 +(cadp / vPFK_KPFKadpa)+(camp / vPFK_KPFKampa)))/(vPFK_KPFKf6ps *(1 +(cpep / vPFK_KPFKpep)+(cadp / vPFK_KPFKadpb)+(camp / vPFK_KPFKampb))))), vPFK_nPFK)))))	[12,28]
Aldo	((cytosol * vALDO_rmaxALDO *(cfdp -((cgap * cdhap)/ vALDO_kALDOeq)))/(vALDO_kALDOfdp + cfdp +((vALDO_kALDOgap * cdhap)/(vALDO_kALDOeq * vALDO_VALDOblf))+((vALDO_kALDOdhap * cgap)/(vALDO_kALDOeq * vALDO_VALDOblf))+((cfdp * cgap)/ vALDO_kALDOgapinh)+((cgap * cdhap)/(vALDO_VALDOblf * vALDO_kALDOeq))))	[12,28]
TpiA	((cytosol * vTIS_rmaxTIS *(cdhap -(cgap / vTIS_kTISeq)))/((vTIS_kTISdhap *(1 +(cgap / vTIS_kTISgap)))+ cdhap))	[12,28]
GapA	((cytosol * vGAPDH_rmaxGAPDH *((cgap * cnad)-((cpgp * cnadh)/ vGAPDH_KGAPDHeq)))/(((vGAPDH_KGAPDHgap *(1 +(cpgp / vGAPDH_KGAPDHpgp)))+ cgap)*((vGAPDH_KGAPDHnad *(1 +(cnadh / vGAPDH_KGAPDHnadh)))+ cnad)))	[12,28]
Pgk	((cytosol * vPGK_rmaxPGK *((cadp * cpgp)-((catp * cpg3)/ vPGK_KPGKeq)))/(((vPGK_KPGKadp *(1 +(catp / vPGK_KPGKatp)))+ cadp)*((vPGK_KPGKpgp *(1 +(cpg3 / vPGK_KPGKpg3)))+ cpgp)))	[12,28]
GpmA	((cytosol * vrpGluMu_rmaxPGluMu *(cpg3 -(cpg2 / vrpGluMu_KPGluMueq)))/((vrpGluMu_KPGluMupg3 *(1 +(cpg2 / vrpGluMu_KPGluMupg2)))+ cpg3))	[12,28]
Eno	((cytosol * vENO_rmaxENO *(cpg2 -(cpep / vENO_KENOeq)))/((vENO_KENOpg2 *(1 +(cpep / vENO_KENOpep)))+ cpg2))	[12,28]
Pyk	((cytosol * vPK_rmaxPK * cpep *pow(((cpep / vPK_KPKpep)+ 1), (vPK_nPK - 1))* cadp)/(vPK_KPKpep *((vPK_LPK *pow(((1 +(catp / vPK_KPKatp))/((cfdp / vPK_KPKfdp)+(camp / vPK_KPKamp)+ 1)), vPK_nPK))+pow(((cpep / vPK_KPKpep)+ 1), vPK_nPK))*(cadp + vPK_KPKadp)))	[12,28]
Ppc	((cytosol * vpepCxylase_rmaxpepCxylase * cpep *(1 +pow((cfdp / vpepCxylase_KpepCxylasefdp), vpepCxylase_npepCxylasefdp)))/(vpepCxylase_KpepCxylasepep + cpep))	[12,28]
Pdh	((cytosol * vPDH_rmaxPDH *pow(cpyr , vPDH_nPDH))/(vPDH_KPDHpyr +pow(cpyr , vPDH_nPDH)))	[12,28]
Pgm	((cytosol * vPGM_rmaxPGM *(cg6p -(cg1p / vPGM_KPGMeq)))/((vPGM_KPGMg6p *(1 +(cg1p / vPGM_KPGMg1p)))+ cg6p))	[12,28]
GlgC	((cytosol * vG1PAT_rmaxG1PAT * cg1p * catp *(1 +pow((cfdp / vG1PAT_KG1PATfdp), vG1PAT_nG1PATfdp)))/((vG1PAT_KG1PATatp + catp)*(vG1PAT_KG1PATg1p + cg1p)))	[12,28]
G6pdh	((cytosol * vG6PDH_rmaxG6PDH * cg6p * cnadp)/((cg6p + vG6PDH_KG6PDHg6p)*(1 +(cnadph / vG6PDH_KG6PDHnadphg6pinh))*((vG6PDH_KG6PDHnadp *(1 +(cnadph / vG6PDH_KG6PDHnadphnadpinh)))+ cnadp)))	[12,28]
Pgdh	((cytosol * vPGDH_rmaxPGDH * cpg * cnadp)/((cpg + vPGDH_KPGDHpg)*(cnadp +(vPGDH_KPGDHnadp *(1 +(cnadph / vPGDH_KPGDHnadphinh))*(1 +(catp / vPGDH_KPGDHatpinh))))))	[12,28]
R5pi	(cytosol * vR5PI_rmaxR5PI *(cribu5p -(crib5p / vR5PI_KR5PIeq)))	[12,28]
Ru5P	(cytosol * vRu5P_rmaxRu5P *(cribu5p -(cxyl5p / vRu5P_KRu5Peq)))	[12,28]
RPPK	((cytosol * vPPK_rmaxRPPK * crib5p)/(vPPK_KRPPKrib5p + crib5p))	[12,28]
Ta	(cytosol * vTA_rmaxTA *((cgap * csed7p)-((ce4p * cf6p)/ vTA_KTAeq)))	[12,28]
Tka	(cytosol * vTKA_rmaxTKa *((crib5p * cxyl5p)-((csed7p * cgap)/ vTKA_KTKaeq)))	[12,28]
Tkb	(cytosol * vTKB_rmaxTKb *((cxyl5p * ce4p)-((cf6p * cgap)/ vTKB_KTKbeq)))	[12,28]
Mursyn	(cytosol * vMURSyNTH_rmaxMurSynth)	[12,28]
Trysyn	(cytosol * vTRPSYNTH_rmaxTrpSynth)	[12,28]
Sersynt	((cytosol * vsersynth_rmaxSerSynth * cpg3)/(vsersynth_KSerSynthpg3 + cpg3))	[12,28]
Synthesis 1	((cytosol * vSynth1_rmaxSynth1 * cpep)/(vSynth1_KSynth1pep + cpep))	[12,28]
Synthesis 2	((cytosol * vSynth2_rmaxSynth2 * cpyr)/(vSynth2_KSynth2pyr + cpyr))	[12,28]
DAHPsyn	((cytosol * vDAHPS_rmaxDAHPS *pow(ce4p , vDAHPS_nDAHPSe4p)*pow(cpep , vDAHPS_nDAHPSpep))/((vDAHPS_KDAHPSe4p +pow(ce4p , vDAHPS_nDAHPSe4p))*(vDAHPS_KDAHPSpep +pow(cpep , vDAHPS_nDAHPSpep))))	[12,28]
Metsyn	(cytosol * vMethSynth_rmaxMetSynth)	[12,28]

Dilu_G3P	((cytosol * vG3PDH_rmaxG3PDH * cdhap)/(vG3PDH_KG3PDHdhap + cdhap))	[12,28]
D_G6P	(cytosol * vG6P_mu * cg6p)	[12,28]
D_F6P	(cytosol * vf6P_mu * cf6p)	[12,28]
D_FDP	(cytosol * vfdP_mu * cfdp)	[12,28]
D_GAP	(cytosol * vGAP_mu * cgap)	[12,28]
D_DHAP	(cytosol * vDHAP_mu * cdhap)	[12,28]
D_PGP	(cytosol * vPGP_mu * cpgp)	[12,28]
D-3PG	(cytosol * vPG3_mu * cpg3)	[12,28]
D_2PG	(cytosol * vpg2_mu * cpg2)	[12,28]
D_PEP	(cytosol * vPEP_mu * cpep)	[12,28]
D_Ru5P	(cytosol * vRibu5p_mu * cribu5p)	[12,28]
D_R5P	(cytosol * vRIB5P_mu * crib5p)	[12,28]
D_Xu5P	(cytosol * vXYL5P_mu * cxyl5p)	[12,28]
D_S7P	(cytosol * vSED7P_mu * csed7p)	[12,28]
D_PYR	(cytosol * vpyr_mu * cpyr)	[12,28]
D_6PG	(cytosol * vPG_mu * cpg)	[12,28]
D_E4P	(cytosol * vE4P_mu * ce4p)	[12,28]
D_G1P	(cytosol * vGLP_mu * cg1p)	[12,28]

Table S2 Places (Initial parameters or equations) of Model-1

Place	Initial parameters or Equation	Source	Place	Initial parameters or Equation	Source
extracellular	1	[12,28]	vTRPSYNTH_rmaxTrpSynth	0.001037	[12,28]
cytosol	1	[12,28]	vG3PDH_rmaxG3PDH	0.01162	[12,28]
cpep	2.67	[12,28]	vG3PDH_KG3PDHdhap	1	[12,28]
cglcex	2	[12,28]	vPGK_rmaxPGK	3021.773771	[12,28]
cg6p	3.48	[12,28]	vPGK_KPGKeq	1934.4	[12,28]
cpyr	2.67	[12,28]	vPGK_KPGKadp	0.185	[12,28]
cf6p	0.6	[12,28]	vPGK_KPGKatp	0.653	[12,28]
cg1p	0.653	[12,28]	vPGK_KPGKpgp	0.0468	[12,28]
cpg	0.808	[12,28]	vPGK_KPGKpg3	0.473	[12,28]
cfdp	0.272	[12,28]	vsersynth_rmaxSerSynth	0.025712	[12,28]
csed7p	0.276	[12,28]	vsersynth_KSerSynthpg3	1	[12,28]
cgap	0.218	[12,28]	vrpGluMu_rmaxPGluMu	89.049654	[12,28]
ce4p	0.098	[12,28]	vrpGluMu_KPGluMueq	0.188	[12,28]
cxyl5p	0.138	[12,28]	vrpGluMu_KPGluMupg3	0.2	[12,28]
crib5p	0.398	[12,28]	vrpGluMu_KPGluMupg2	0.369	[12,28]
cdhap	0.167	[12,28]	vENO_rmaxENO	330.447615	[12,28]
cpgp	0.008	[12,28]	vENO_KENOeq	6.73	[12,28]
cpg3	2.13	[12,28]	vENO_KENOpg2	0.1	[12,28]
cpg2	0.399	[12,28]	vENO_KENOpep	0.135	[12,28]
cribu5p	0.111	[12,28]	vPK_rmaxPK	0.061132	[12,28]
vPTS_rmaxPTS	7829.78	[12,28]	vPK_KPKpep	0.31	[12,28]
vPTS_KPTSa1	3082.3	[12,28]	vPK_nPK	4	[12,28]
vPTS_KPTSa2	0.01	[12,28]	vPK_LPK	1000	[12,28]
vPTS_KPTSa3	245.3	[12,28]	vPK_KPKatp	22.5	[12,28]
vPTS_nPTSg6p	3.66	[12,28]	vPK_KPKfdp	0.19	[12,28]
vPTS_KPTSg6p	2.15	[12,28]	vPK_KPKamp	0.2	[12,28]
vPGI_rmaxPGI	650.987869	[12,28]	vPK_KPKadp	0.26	[12,28]
vPGI_KPGIeq	0.1725	[12,28]	vpepCxylase_rmaxpepCxylase	0.107021	[12,28]
vPGI_KPGIg6p	2.9	[12,28]	vpepCxylase_KpepCxylasefdp	0.7	[12,28]
vPGI_KPGIf6p	0.266	[12,28]	vpepCxylase_npepCxylasefdp	4.21	[12,28]
vPGI_KPGIf6ppginh	0.2	[12,28]	vpepCxylase_KpepCxylasepep	4.07	[12,28]
vPGI_KPGIg6ppginh	0.2	[12,28]	vSynth1_rmaxSynth1	0.019539	[12,28]
vPGM_rmaxPGM	0.839824	[12,28]	vSynth1_KSynth1pep	1	[12,28]
vPGM_KPGMeq	0.196	[12,28]	vSynth2_rmaxSynth2	0.073619	[12,28]
vPGM_KPGMg6p	1.038	[12,28]	vSynth2_KSynth2pyr	1	[12,28]
vPGM_KPGMg1p	0.0136	[12,28]	vDAHPS_rmaxDAHPS	0.107953	[12,28]
vG6PDH_rmaxG6PDH	1.380197	[12,28]	vDAHPS_nDAHPSe4p	2.6	[12,28]
vG6PDH_KG6PDHg6p	14.4	[12,28]	vDAHPS_nDAHPSpep	2.2	[12,28]
vG6PDH_KG6PDHnadphg6pinh	6.43	[12,28]	vDAHPS_KDAHPSe4p	0.035	[12,28]
vG6PDH_KG6PDHnadp	0.0246	[12,28]	vDAHPS_KDAHPSpep	0.0053	[12,28]
vG6PDH_KG6PDHnadphnadpinh	0.01	[12,28]	vPDH_rmaxPDH	6.059531	[12,28]
vPFK_rmaxPFK	1840.584747	[12,28]	vPDH_nPDH	3.68	[12,28]
vPFK_KPFKatps	0.123	[12,28]	vPDH_KPDHpyr	1159	[12,28]
vPFK_KPFKadpc	4.14	[12,28]	vMethSynth_rmaxMetSynth	0.002263	[12,28]
vPFK_KPFKf6ps	0.325	[12,28]	vPGDH_rmaxPGDH	16.23236	[12,28]
vPFK_KPFKpep	3.26	[12,28]	vPGDH_KPGDHpg	37.5	[12,28]
vPFK_KPFKadpb	3.89	[12,28]	vPGDH_KPGDHnadp	0.0506	[12,28]
vPFK_KPFKampb	3.2	[12,28]	vPGDH_KPGDHnadphinh	0.0138	[12,28]
vPFK_KPFKadpa	128	[12,28]	vPGDH_KPGDHatpinh	208	[12,28]
vPFK_KPFKampa	19.1	[12,28]	vR5PI_rmaxR5PI	4.838412	[12,28]
vPFK_LPFK	5.63E+06	[12,28]	vR5PI_KR5PIeq	4	[12,28]
vPFK_nPFK	11.1	[12,28]	vRu5P_rmaxRu5P	6.739029	[12,28]
vTA_rmaxTA	10.871641	[12,28]	vRu5P_KRu5Peq	1.4	[12,28]
vTA_KTAeq	1.05	[12,28]	vPPK_rmaxRPPK	0.0129	[12,28]
vTKA_rmaxTKa	9.473385	[12,28]	vPPK_KRPPKrib5p	0.1	[12,28]
vTKA_KTKaeq	1.2	[12,28]	vG1PAT_rmaxG1PAT	0.007525	[12,28]
vTKB_rmaxTKb	86.558559	[12,28]	vG1PAT_KG1PATfdp	0.119	[12,28]
vTKB_KTKbeq	10	[12,28]	vG1PAT_nG1PATfdp	1.2	[12,28]
vMURSyNTH_rmaxMurSynth	0.000437	[12,28]	vG1PAT_KG1PATatp	4.42	[12,28]
vALDO_rmaxALDO	17.414644	[12,28]	vG1PAT_KG1PATg1p	3.2	[12,28]
vALDO_kALDOeq	0.144	[12,28]	vG6P_mu	0.000028	[12,28]

vALDO_kALDOfdp	1.75	[12,28]	vf6P_mu	0.000028	[12,28]
vALDO_kALDOgap	0.088	[12,28]	vfdP_mu	0.000028	[12,28]
vALDO_VALDOblf	2	[12,28]	vGAP_mu	0.000028	[12,28]
vALDO_kALDOdhap	0.088	[12,28]	vDHAP_mu	0.000028	[12,28]
vALDO_kALDOgapinh	0.6	[12,28]	vPGP_mu	0.000028	[12,28]
vGAPDH_rmaxGAPDH	921.594286	[12,28]	vPG3_mu	0.000028	[12,28]
vGAPDH_KGAPDHeq	0.63	[12,28]	vpg2_mu	0.000028	[12,28]
vGAPDH_KGAPDHgap	0.683	[12,28]	vPEP_mu	0.000028	[12,28]
vGAPDH_KGAPDHpgp	0.00001	[12,28]	vRibu5p_mu	0.000028	[12,28]
vGAPDH_KGAPDHnad	0.252	[12,28]	vRIB5P_mu	0.000028	[12,28]
vGAPDH_KGAPDHnadh	1.09	[12,28]	vXYL5P_mu	0.000028	[12,28]
vTIS_rmaxTIS	68.674744	[12,28]	vSED7P_mu	0.000028	[12,28]
vTIS_kTISeq	1.39	[12,28]	vpyr_mu	0.000028	[12,28]
vTIS_kTISdhap	2.8	[12,28]	vPG_mu	0.000028	[12,28]
vTIS_kTISgap	0.3	[12,28]	vE4P_mu	0.000028	[12,28]
vEXTER_cfeed	110.96	[12,28]	vGLP_mu	0.000028	[12,28]
vEXTER_Dil	0.000028	[12,28]			[12,28]
catp	(4.27 -(4.163 *(t /(0.657 +(1.43 * t)+(0.0364 *pow(t , 2))))))				[12,28]
cadp	(0.582 +(1.73 *pow(2.731 , (-0.15 * t))*((0.12 * t)+(0.000214 *pow(t , 3)))))				[12,28]
camp	(0.123 +(7.25 *(t /(7.25 +(1.47 * t)+(0.17 *pow(t , 2)))))+(1.073 /(1.29 +(8.05 * t))))				[12,28]
cnadp	((0.159 -(0.00554 *(t /((2.8 -(0.271 * t))+(0.01 *pow(t , 2))))))+(0.182 /(4.82 +(0.526 * t))))				[12,28]
cnadph	(0.062 +(0.332 *pow(2.718 , (-0.464 * t))*((0.0166 *pow(t , 1.58))+(0.000166 *pow(t , 4.73))+(0.1312 *pow(10 , -9)*pow(t , 7.89))+(0.1362 *pow(10 , -12)*pow(t , 11))+(0.1233 *pow(10 , -15)*pow(t , 14.2)))))				[12,28]
cnad	((1.314 +(1.314 *pow(2.73 , ((-0.0435 * t)- 0.342))))-((t + 7.871)*(pow(2.73 , ((-0.0218 * t)- 0.171))/(8.481 + t))))				[12,28]
cnadh	(0.0934 +(0.00111 *pow(2.371 , (-0.123 * t))*((0.844 * t)+(0.104 *pow(t , 3)))))				[12,28]
t	getElapsedTime(simulator)				[12,28]

Table S3 Equations of Model-2.

Process	Kinetic Script	Source	Process	Kinetic Script	Source
k10	k10*GlcPEIICB*k	[13]	k-10	k010*EIICB*G6P*k	[13]
k9	k9*PEIICB*Glc*k	[13]	k-9	k09*GlcPEIICB*k	[13]
k8	k8*EIICBPEIIA*k	[13]	k-8	k08*EIIA*PEIICB*k	[13]
k7	k7*PEIIA*EIICB*k	[13]	k-7	k07*EIICBPEIIA*k	[13]
k6	k6*EIIAPHpr*k	[13]	k-6	k06*Hpr*PEIIA*k	[13]
k5	k5*PHpr*EIIA*k	[13]	k-5	k05*EIIAPHpr*k	[13]
k4	k4*HprPEI*k	[13]	k-4	k04*EI*PHpr*k	[13]
k3	k3*PEI*Hpr*k	[13]	k-3	k03*HprPEI*k	[13]
k2	k2*EIPEP*k	[13]	k-2	k02*PYR*PEI*k	[13]
k1	k1*PEP*EI*k	[13]	k-1	k01*EIPEP*k	[13]
D	Glc*D6	[13]	D	Hpr*D3	[13]
D	PEIICB*D0	[13]	D	PEI*D1	[13]
D	EIIA*D5	[13]	D	PYR*D6	[13]
D	PHpr*D3	[13]	D	GlcPEIICB*D6	[13]
D	EI*D1	[13]	D	EIICBPEIIA*D0	[13]
D	PEP*D6	[13]	D	EIIAPHpr*D4	[13]
D	G6P*D6	[13]	D	HprPEI*D2	[13]
D	EIICB*D0	[13]	D	EIPEP*D1	[13]
D	PEIIA*D5	[13]			

Table S4 Different sources initial values of PTS enzymes of Model-2.

Enzyme	Initial Value from [13] Concentration (μM)	Initial Value from [9] Molecule number	Initial Value of this work Concentration (μM)
EI	5	1577	1.2
HPr	50	15766	1.1
EIIA	40	12623	0.65
EIICB	6.7	2100	0.1
PEI	0	0	0.6
PHPr	0	0	28
PEIIA	0	0	21
PEIICB	0	0	5.6
EI::PEP	0	0	2.4
PEI::HPr	0	0	0.7
PHPr::EIIA	0	0	18
PEIIA::EIICB	0	0	1.25
PEIICB::Glc	0	0	0
Glucose	500	70767	500
G6P	50	15766	50
PEP	2800	882890	2800
PYR	900	283780	900

Table S5 Parameters of Model-2.

Variable	Initial Value	Source	Variable	Initial Value	Source
EI	1.2	This study	k1	1960	[13]
Hpr	1.1	This study	k01 (k-1)	480000	[13]
EIIA	0.65	This study	k2	108000	[13]
EIICB	0.1	This study	k02 (k-2)	294	[13]
PEI	0.6	This study	k3	14000	[13]
PHpr	28	This study	k03 (k-3)	14000	[13]
PEIIA	21	This study	k4	84000	[13]
PEIICB	5.6	This study	k04 (k-4)	3360	[13]
PEP	2800	[13]	k5	21960	[13]
PYR	900	[13]	k05 (k-5)	21960	[13]
Glcucose	500	[13]	k6	4392	[13]
G6P	50	[13]	k06 (k-6)	3384	[13]
EIPEP	2.4	[13]	k7	880	[13]
HprPEI	0.7	[13]	k07 (k-7)	880	[13]
EIIAPHpr	18	[13]	k8	2640	[13]
EIICBPEIIA	1.25	[13]	k08 (k-8)	960	[13]
GlcPEIICB	0	[13]	k9	260	[13]
D0	0*d	[13]	k09 (k-9)	389	[13]
D1	197.8*d	[13]	k10	4800	[13]
D2	189.1*d	[13]	k010 (k-10)	0.0054	[13]
D3	378*d	[13]			
D4	262.1*d	[13]			
D5	300*d	[13]			
D6	18000*d	[13]			
d	0.0001	This study			
k	1	This study			

Table S6 Equations of Model-4.

Name	Kinetic Script	Pathway#	Source
k-1	(k01 * EIPEP) * k	PTS	[13] & this study
k-10	((k010 * EIICB) * cg6p) * k	PTS	[13] & this study
k-2	((k02 * cpyr) * PEI) * k	PTS	[13] & this study
k-3	(k03 * HprPEI) * k	PTS	[13] & this study
k-4	((k04 * EI) * PHpr) * k	PTS	[13] & this study
k-5	(k05 * EIIAPHpr) * k	PTS	[13] & this study
k-6	((k06 * Hpr) * PEIIA) * k	PTS	[13] & this study
k-7	(k07 * EIICBPEIIA) * k	PTS	[13] & this study
k-8	((k08 * EIIA) * PEIICB) * k	PTS	[13] & this study
k-9	(k09 * GLUPEIICB) * k	PTS	[13] & this study
k1	((k1 * cpep) * EIEI) * k	PTS	[13] & this study
k10	(k10 * GLUPEIICB) * k	PTS	[13] & this study
k2	(k2 * EIPEP) * k	PTS	[13] & this study
k3	((k3 * PEI) * Hpr) * k	PTS	[13] & this study
k4	(k4 * HprPEI) * k	PTS	[13] & this study
k5	((k5 * PHpr) * EIIA) * k	PTS	[13] & this study
k6	(k6 * EIIAPHpr) * k	PTS	[13] & this study
k7	((k7 * PEIIA) * EIICB) * k	PTS	[13] & this study
k8	(k8 * EIICBPEIIA) * k	PTS	[13] & this study
k9	((k9 * PEIICB) * Glc) * k	PTS	[13] & this study
ptsG	1/(cfdp+0.1)/10 * exp	PTS	this study
ptsHIcrr	cfdp*exp	PTS	this study
G3PDH	((cytosol * vGAPDH_rmaxGAPDH) * ((cgap * cnad) - ((cpgp * cnadh) / vGAPDH_KGAPDHeq))) / (((vGAPDH_KGAPDHgap * (1 + (cpgp / vGAPDH_KGAPDHpgp))) + cgap) * ((vGAPDH_KGAPDHnad * (1 + (cnadh / vGAPDH_KGAPDHnadh))) + cnad))	PPP	[12,28]
G6PDH	(((cytosol * vG6PDH_rmaxG6PDH) * cg6p) * cnadp) / (((cg6p + vG6PDH_KG6PDHg6p) * (1 + (cnadph / vG6PDH_KG6PDHnadphg6pinh))) * ((vG6PDH_KG6PDHnadp * (1 + (cnadph / vG6PDH_KG6PDHnadphnadpinh))) + cnadp))	PPP	[12,28]
PGDH	(((cytosol * vPGDH_rmaxPGDH) * cpg) * cnadp) / ((cpg + vPGDH_KPGDHpg) * (cnadp + ((vPGDH_KPGDHnadp * (1 + (cnadph / vPGDH_KPGDHnadphinh))) * (1 + (catp / vPGDH_KPGDHatpinh)))))	PPP	[12,28]
R5PI	(cytosol * vR5PI_rmaxR5PI) * (cribu5p - (crib5p / vR5PI_KR5PIeq))	PPP	[12,28]
RPPK	((cytosol * vPPK_rmaxRPPK) * crib5p) / (vPPK_KRPPKrib5p + crib5p)	PPP	[12,28]
Ru5P	(cytosol * vRu5P_rmaxRu5P) * (cribu5p - (cxyl5p / vRu5P_KRu5Peq))	PPP	[12,28]
Ta	(cytosol * vTA_rmaxTA) * ((cgap * csed7p) - ((ce4p * cf6p) / vTA_KTAeq))	PPP	[12,28]
TkA	(cytosol * vTKA_rmaxTKa) * ((crib5p * cxyl5p) - ((csed7p	PPP	[12,28]

	* cgap) / vTKA_KTKaeq))		
TktB	(cytosol * vTKB_rmaxTKb) * ((cxyl5p * ce4p) - ((cf6p * cgap) / vTKB_KTKbeq))	PPP	[12,28]
(P)HPr::GlgP	glg*(Hpr/(0.00001+PHpr))*f1	GLG	[12,28]
AspP	ADPG*0.1	GLG	[12,28]
GlgA	ADPG*f2*glgCA	GLG	[12,28]
GlgC	G1P*f2*glgCA	GLG	[12,28]
PGM _backward	G1P*1.0	GLG	[12,28]
PGM _foreward	cg6p*1.0	GLG	[12,28]
ALDP	((cytosol * vALDO_rmaxALDO) * (cfdp - ((cgap * cdhap) / vALDO_kALDOeq))) / (((((vALDO_kALDOfdp + cfdp) + ((vALDO_kALDOgap * cdhap) / (vALDO_kALDOeq * vALDO_VALDOblf))) + ((vALDO_kALDOdhap * cgap) / (vALDO_kALDOeq * vALDO_VALDOblf))) + ((cfdp * cgap) / vALDO_kALDOgapinh)) + ((cgap * cdhap) / (vALDO_VALDOblf * vALDO_kALDOeq)))	EMD	[12,28]
DAHPsyn	(((cytosol * vDAHPS_rmaxDAHPS) * pow(ce4p, vDAHPS_nDAHPSe4p)) * pow(cpep, vDAHPS_nDAHPSpep)) / ((vDAHPS_KDAHPSe4p + pow(ce4p, vDAHPS_nDAHPSe4p)) * (vDAHPS_KDAHPSpep + pow(cpep, vDAHPS_nDAHPSpep)))	EMD	[12,28]
ENO	((cytosol * vENO_rmaxENO) * (cpg2 - (cpep / vENO_KENOeq))) / ((vENO_KENOpg2 * (1 + (cpep / vENO_KENOpep))) + cpg2)	EMD	[12,28]
Metsyn	cytosol * vMethSynth_rmaxMetSynth	EMD	[12,28]
Mursyn	cytosol * vMURSyNTH_rmaxMurSynth	EMD	[12,28]
PDH	((cytosol * vPDH_rmaxPDH) * pow(cpyr, vPDH_nPDH)) / (vPDH_KPDHpyr + pow(cpyr, vPDH_nPDH))	EMD	[12,28]
PFK	(((cytosol * vPFK_rmaxPFK) * catp) * cf6p) / (((catp + (vPFK_KPFKatps * (1 + (cadp / vPFK_KPFKadpc)))) * (cf6p + ((vPFK_KPFKf6ps * (((1 + (cpep / vPFK_KPFKpep)) + (cadp / vPFK_KPFKadpb)) + (camp / vPFK_KPFKampb))) / ((1 + (cadp / vPFK_KPFKadpa)) + (camp / vPFK_KPFKampa))))) * (1 + (vPFK_LPFK / pow(1 + ((cf6p * ((1 + (cadp / vPFK_KPFKadpa)) + (camp / vPFK_KPFKampa))) / (vPFK_KPFKf6ps * (((1 + (cpep / vPFK_KPFKpep)) + (cadp / vPFK_KPFKadpb)) + (camp / vPFK_KPFKampb)))), vPFK_nPFK))))	EMD	[12,28]
PGI	((cytosol * vPGI_rmaxPGI) * (cg6p - (cf6p / vPGI_KPGIeq))) / ((vPGI_KPGIg6p * ((1 + (cf6p / (vPGI_KPGIf6p * (1 + (cpg / vPGI_KPGIf6ppginh))))) + (cpg / vPGI_KPGIg6ppginh))) + cg6p)	EMD	[12,28]
PGK	((cytosol * vPGK_rmaxPGK) * ((cadp * cpgp) - ((catp * cpg3) / vPGK_KPGKeq))) / (((vPGK_KPGKadp * (1 + (catp / vPGK_KPGKatp))) + cadp) * ((vPGK_KPGKpgp * (1 + (cpg3 / vPGK_KPGKpg3))) + cpgp))	EMD	[12,28]
PGLM	((cytosol * vrpGluMu_rmaxPGluMu) * (cpg3 - (cpg2 / vrpGluMu_KPGluMueq))) / ((vrpGluMu_KPGluMupg3 * (1 + (cpg2 / vrpGluMu_KPGluMupg2))) + cpg3)	EMD	[12,28]
PK	((((cytosol * vPK_rmaxPK) * cpep) * pow((cpep / vPK_KPKpep) + 1, vPK_nPK - 1)) * cadp) / ((vPK_KPKpep * ((vPK_LPK * pow((1 + (catp / vPK_KPKatp)) / (((cfdp / vPK_KPKfdp) + (camp / vPK_KPKamp)) + 1), vPK_nPK)) + pow((cpep / vPK_KPKpep) + 1, vPK_nPK))) * (cadp + vPK_KPKadp))	EMD	[12,28]
PPC	(((cytosol * vpepCxylase_rmaxpepCxylase) * cpep) * (1 + pow(cfdp / vpepCxylase_KpepCxylasefdp, vpepCxylase_npepCxylasefdp))) / (vpepCxylase_KpepCxylasepep + cpep)	EMD	[12,28]
Sersyn	((cytosol * vsersynth_rmaxSerSynth) * cpg3) / (vsersynth_KSerSynthpg3 + cpg3)	EMD	[12,28]
Syn 1	((cytosol * vSynth1_rmaxSynth1) * cpep) /	EMD	[12,28]

	(vSynth1_KSynth1pep + cpep)		
Syn 2	((cytosol * vSynth2_rmaxSynth2) * cpyr) / (vSynth2_KSynth2pyr + cpyr)	EMD	[12,28]
TIS	((cytosol * vTIS_rmaxTIS) * (cdhap - (cgap / vTIS_kTISeq))) / ((vTIS_kTISdhap * (1 + (cgap / vTIS_kTISgap))) + cdhap)	EMD	[12,28]
Trysyn	cytosol * vTRPSYNTH_rmaxTrpSynth	EMD	[12,28]
D	G1P * u	D&D	this study
D	ADPG* u	D&D	this study
D	PHpr * D3	D&D	[13]
D	EIIA * D5	D&D	[13]
D	PEIICB * D0	D&D	[13]
D	PEIIA * D5	D&D	[13]
D	EI * D1	D&D	[13]
D	Hpr * D3	D&D	[13]
D	EIICB * D0	D&D	[13]
D	PEI * D1	D&D	[13]
D	GLUPEIICB * D0	D&D	[13]
D	EIIAPHpr * D4	D&D	[13]
D	EIICBPEIIA * D0	D&D	[13]
D	HprPEI * D2	D&D	[13]
D	EIPEP * D1	D&D	[13]
D	EIEI * D1	D&D	[13]
D	(cytosol * vSED7P_mu) * csed7p	D&D	[12,28]
D	(cytosol * vPEP_mu) * cpep	D&D	[12,28]
D	(cytosol * vDHAP_mu) * cdhap	D&D	[12,28]
D	(cytosol * vpyr_mu) * cpyr	D&D	[12,28]
D	(cytosol * vRibu5p_mu) * cribu5p	D&D	[12,28]
D	(cytosol * vPG3_mu) * cpg3	D&D	[12,28]
D	(cytosol * vRIB5P_mu) * crib5p	D&D	[12,28]
D	(cytosol * vG6P_mu) * cg6p	D&D	[12,28]
D	(cytosol * vXYL5P_mu) * cxyl5p	D&D	[12,28]
D	(cytosol * vPG_mu) * cpg	D&D	[12,28]
D	(cytosol * vf6P_mu) * cf6p	D&D	[12,28]
D	(cytosol * vfdP_mu) * cfdp	D&D	[12,28]
D	(cytosol * vPGP_mu) * cpgp	D&D	[12,28]
D	(cytosol * vGAP_mu) * cgap	D&D	[12,28]
D	(cytosol * vpg2_mu) * cpg2	D&D	[12,28]
D	(cytosol * vE4P_mu) * ce4p	D&D	[12,28]
D	((cytosol * vG3PDH_rmaxG3PDH) * cdhap) / (vG3PDH_KG3PDHdhap + cdhap)	D&D	[12,28]
DIMER	if (Z6==1) {EI*cpep*r3} else if (Z5==1) {EI*cpep*0.0001} else if (Z3==1) {EI*cpep*r2} else {EI*cpep*r1}	CON	this study
G_cAMP	PEIIA*0.1	CON	this study
G_PTSE	PTSall*0.1	CON	this study
Out_pole	pole / glg	CON	this study
Out_scatter	scatter*0.3	CON	this study
Out-cAMP	cycAMP*1.0	CON	this study
To_pole	(glg * 0.1) / scatter	CON	this study
To_scatter	Glc*0.1	CON	this study
X3	X3	CON	this study
X5	X5	CON	this study
X6	X6	CON	this study
Z3	Z3	CON	this study
Z5	Z5	CON	this study
Z6	Z6	CON	this study

Abbrevation of pathway: EMD (glycolysis), PPP (pentose phosphate pathway), CON (control mechanisms), GLG (glycogen metabolism pathway), PTS (PTS system), D&D (degradation or dilusion)

Table S7 Parameters of Model-4.

Variable	Initial Value	Source	Variable	Initial Value	Source
D0	0*d	[13]	cytosol	1	[12,28]
D1	197.8*d	[13]	extracellular	1	[12,28]
D2	189.1*d	[13]	vALDO_kALDOdhap	0.088	[12,28]
D3	378*d	[13]	vALDO_kALDOeq	0.144	[12,28]
D4	262.1*d	[13]	vALDO_kALDOfdp	1.75	[12,28]
D5	300*d	[13]	vALDO_kALDOgap	0.088	[12,28]
EI	1.2*cc	this study	vALDO_kALDOgapinh	0.6	[12,28]
EIEI	0	this study	vALDO_rmaxALDO	17.414644	[12,28]
EIIA	0.65*cc	this study	vALDO_VALDOblf	2	[12,28]
EIIAPHpr	18*cc	this study	vDAHPS_KDAHPSe4p	0.035	[12,28]
EIICB	0.1*cc	this study	vDAHPS_KDAHPSpep	0.0053	[12,28]
EIICBPEIIA	1.25*cc	this study	vDAHPS_nDAHPSe4p	2.6	[12,28]
EIPEP	2.4*cc	this study	vDAHPS_nDAHPSpep	2.2	[12,28]
GLUPEIICB	0	this study	vDAHPS_rmaxDAHPS	0.107953	[12,28]
Hpr	1.1*cc	this study	vDHAP_mu	0.000028	[12,28]
HprPEI	0.7*cc	this study	vE4P_mu	0.000028	[12,28]
k01	480000	[13]	vENO_KENOeq	6.73	[12,28]
k010	0.0054	[13]	vENO_KENOpep	0.135	[12,28]
k02	294	[13]	vENO_KENOpg2	0.1	[12,28]
k03	14000	[13]	vENO_rmaxENO	330.447615	[12,28]
k04	3360	[13]	vEXTER_cfeed	110.96	[12,28]
k05	21960	[13]	vEXTER_Dil	0.000028	[12,28]
k06	3384	[13]	vf6P_mu	0.000028	[12,28]
k07	880	[13]	vfdP_mu	0.000028	[12,28]
k08	960	[13]	vG1PAT_KG1PATatp	4.42	[12,28]
k09	389	[13]	vG1PAT_KG1PATfdp	0.119	[12,28]
k1	1960	[13]	vG1PAT_KG1PATg1p	3.2	[12,28]
k10	4800	[13]	vG1PAT_nG1PATfdp	1.2	[12,28]
k2	108000	[13]	vG1PAT_rmaxG1PAT	0.007525	[12,28]
k3	14000	[13]	vG3PDH_KG3PDHdhap	1	[12,28]
k4	84000	[13]	vG3PDH_rmaxG3PDH	0.01162	[12,28]
k5	21960	[13]	vG6P_mu	0.000028	[12,28]
k6	4392	[13]	vG6PDH_KG6PDHg6p	14.4	[12,28]
k7	880	[13]	vG6PDH_KG6PDHnadp	0.0246	[12,28

]

k8	2640	[13]	vG6PDH_KG6PDHnadphg6pinh	6.43	[12,28]
k9	260	[13]	vG6PDH_KG6PDHnadphnadpinh	0.01	[12,28]
PEI	0.6*cc	this study	vG6PDH_rmaxG6PDH	1.380197	[12,28]
PEIIA	21*cc	this study	vGAP_mu	0.000028	[12,28]
PEIICB	5.6*cc	this study	vGAPDH_KGAPDHeq	0.63	[12,28]
PHpr	28*cc	this study	vGAPDH_KGAPDHgap	0.683	[12,28]
ce4p	0.098	[12,28]	vGAPDH_KGAPDHnad	0.252	[12,28]
crib5p	0.398	[12,28]	vGAPDH_KGAPDHnadh	1.09	[12,28]
cribu5p	0.111	[12,28]	vGAPDH_KGAPDHpgp	0.00001	[12,28]
csed7p	0.276	[12,28]	vGAPDH_rmaxGAPDH	921.594286	[12,28]
cxyl5p	0.138	[12,28]	vGLP_mu	0.000028	[12,28]
ADPG	0.1	this study	vMethSynth_rmaxMetSynth	0.002263	[12,28]
G1P	0.653	this study	vMURSyNTH_rmaxMurSynth	0.000437	[12,28]
glycogen	17	this study	vPDH_KPDHpyr	1159	[12,28]
cf6p	0.6	[12,28]	vPDH_nPDH	3.68	[12,28]
cfdp	0.272	[12,28]	vPDH_rmaxPDH	6.059531	[12,28]
cg6p	3.48	[12,28]	vPEP_mu	0.000028	[12,28]
cgap	0.218	[12,28]	vpepCxylase_KpepCxylasefdp	0.7	[12,28]
cpep	2.67	[12,28]	vpepCxylase_KpepCxylasepep	4.07	[12,28]
cpg	0.808	[12,28]	vpepCxylase_npepCxylasefdp	4.21	[12,28]
cpg2	0.399	[12,28]	vpepCxylase_rmaxpepCxylase	0.107021	[12,28]
cpg3	2.13	[12,28]	vPFK_KPFKadpa	128	[12,28]
cpgp	0.008	[12,28]	vPFK_KPFKadpb	3.89	[12,28]
cpyr	2.67	[12,28]	vPFK_KPFKadpc	4.14	[12,28]
Glcucose	17	this study	vPFK_KPFKampa	19.1	[12,28]
c	1	this study	vPFK_KPFKampb	3.2	[12,28]
cc	0.001	this study	vPFK_KPFKatps	0.123	[12,28]
cycAMP	0.1	this study	vPFK_KPFKf6ps	0.325	[12,28]
d	0.0001	this study	vPFK_KPFKpep	3.26	[12,28]
exp	0.05	this study	vPFK_LPFK	5.63E+06	[12,28]
f1	0.05	this study	vPFK_nPFK	11.1	[12,28]
f2	2	this study	vPFK_rmaxPFK	1840.584747	[12,28]
m2	0	this study	vPG_mu	0.000028	[12,28]
n	1	this study	vpg2_mu	0.000028	[12,28]
nb	0.28	this study	vPG3_mu	0.000028	[12,28]

pole	0.01	this study	vPGDH_KPGDHatpinh	208	[12,28]
PTSE	1	this study	vPGDH_KPGDHnadp	0.0506	[12,28]
r	1.7	this study	vPGDH_KPGDHnadphinh	0.0138	[12,28]
r1	0.001	this study	vPGDH_KPGDHpg	37.5	[12,28]
r2	15	this study	vPGDH_rmaxPGDH	16.23236	[12,28]
r3	50	this study	vPGI_KPGIeq	0.1725	[12,28]
r4	2	this study	vPGI_KPGIf6p	0.266	[12,28]
ratio	cpep/cpyr	this study	vPGI_KPGIf6ppginh	0.2	[12,28]
s	0.3	this study	vPGI_KPGIg6p	2.9	[12,28]
s1	1.2	this study	vPGI_KPGIg6ppginh	0.2	[12,28]
s2	200	this study	vPGI_rmaxPGI	650.987869	[12,28]
s3	0.1	this study	vPGK_KPGKadp	0.185	[12,28]
scatter	0	this study	vPGK_KPGKatp	0.653	[12,28]
u	0.000028	this study	vPGK_KPGKeq	1934.4	[12,28]
v	40	this study	vPGK_KPGKpg3	0.473	[12,28]
X3	1	this study	vPGK_KPGKpgp	0.0468	[12,28]
X5	1	this study	vPGK_rmaxPGK	3021.773771	[12,28]
X6	1	this study	vPGM_KPGMeq	0.196	[12,28]
Z3	0	this study	vPGM_KPGMg1p	0.0136	[12,28]
Z5	0	this study	vPGM_KPGMg6p	1.038	[12,28]
Z6	0	this study	vPGM_rmaxPGM	0.839824	[12,28]
vPGP_mu	0.000028	[12,28]	vRu5P_rmaxRu5P	6.739029	[12,28]
vPK_KPKadp	0.26	[12,28]	vSED7P_mu	0.000028	[12,28]
vPK_KPKamp	0.2	[12,28]	vsersynth_KSerSynthpg3	1	[12,28]
vPK_KPKatp	22.5	[12,28]	vsersynth_rmaxSerSynth	0.025712	[12,28]
vPK_KPKfdp	0.19	[12,28]	vSynth1_KSynth1pep	1	[12,28]
vPK_KPKpep	0.31	[12,28]	vSynth1_rmaxSynth1	0.019539	[12,28]
vPK_LPK	1000	[12,28]	vSynth2_KSynth2pyr	1	[12,28]
vPK_nPK	4	[12,28]	vSynth2_rmaxSynth2	0.073619	[12,28]
vTA_KTAeq	1.05	[12,28]	vPK_rmaxPK	0.061132	[12,28]
vTA_rmaxTA	10.871641	[12,28]	vPPK_KRPPKrib5p	0.1	[12,28]
vTIS_kTISdhap	2.8	[12,28]	vPPK_rmaxRPPK	0.0129	[12,28]
vTIS_kTISeq	1.39	[12,28]	vrpGluMu_KPGluMupg2	0.369	[12,28]
vTIS_kTISgap	0.3	[12,28]	vrpGluMu_KPGluMupg3	0.2	[12,28]
vTIS_rmax	68.674744	[12,28]	vrpGluMu_rmaxPGluMu	89.049654	[12,28

TIS					]
vTKA_KTKaeq	1.2	[12,28]	vRu5P_KRu5Peq	1.4	[12,28]
vTKA_rmaxTKa	9.473385	[12,28]	vPTS_KPTSg6p	2.15	[12,28]
vTKB_KTKbeq	10	[12,28]	vPTS_nPTSg6p	3.66	[12,28]
vTKB_rmaxTKb	86.558559	[12,28]	vPTS_rmaxPTS	7829.78	[12,28]
vpyr_mu	0.000028	[12,28]	vTRPSYNTH_rmaxTrpSynth	0.001037	[12,28]
vXYL5P_mu	0.000028	[12,28]	vR5PI_KR5PIeq	4	[12,28]
vPTS_KPTSa1	3082.3	[12,28]	vR5PI_rmaxR5PI	4.838412	[12,28]
vPTS_KPTSa2	0.01	[12,28]	vRIB5P_mu	0.000028	[12,28]
vPTS_KPTSa3	245.3	[12,28]	vRibu5p_mu	0.000028	[12,28]
			vrpGluMu_KPGluMueq	0.188	[12,28]

bool_cAMP	if (cycAMP>0.05){1} else {0}	this study
bool_FDP	if (cfdp>1.5){1} else{0}	this study
glgCA	if (bool_FDP==1){if(bool_cAMP==1){1} else{0.5}} else {0.01}	this study
k	if (Z6==1) { if (Glc>4){s2} else {0.1}} else {s1}	this study
m	getElapsedTime(simulator)	this study
PTSall	EI+EIEI+Hpr+EIIA+EIICB+PEI+PHpr+PEIIA+PEIICB+EIPEP+HprPEI+EIIAPHpr+EIICBPEIIA+GLUPEIICB	this study
t	getElapsedTime(simulator)	this study
cadp	(0.582 +(1.73 *pow(2.731 , (-0.15 * t))*((0.12 * t)+(0.000214 *pow(t , 3)))))	[12,28]
camp	(0.123 +(7.25 *(t /(7.25 +(1.47 * t)+(0.17 *pow(t , 2)))))+(1.073 /(1.29 +(8.05 * t))))	[12,28]
catp	(4.27 -(4.163 *(t /(0.657 +(1.43 * t)+(0.0364 *pow(t , 2))))))	[12,28]
cdhap	0.167	[12,28]
cnad	((1.314 +(1.314 *pow(2.73 , ((-0.0435 * t)- 0.342))))-((t + 7.871)*(pow(2.73 , ((-0.0218 * t)- 0.171))/(8.481 + t))))	[12,28]
cnadh	(0.0934 +(0.00111 *pow(2.371 , (-0.123 * t))*((0.844 * t)+(0.104 *pow(t , 3)))))	[12,28]
cnadp	((0.159 -(0.00554 *(t /((2.8 -(0.271 * t))+(0.01 *pow(t , 2))))))+(0.182 /(4.82 +(0.526 * t))))	[12,28]
cnadph	(0.062 +(0.332 *pow(2.718 , (-0.464 * t))*((0.0166 *pow(t , 1.58))+(0.000166 *pow(t , 4.73))+(0.1312 *pow(10 , -9)*pow(t , 7.89))+(0.1362 *pow(10 , -12)*pow(t , 11))+(0.1233 *pow(10 , -15)*pow(t , 14.2)))))	[12,28]

Table S8 Arcs' firing value of Model-4.

Source Place	Target Transition	Firing value
scatter	k7	2.5
pole	(P)HPr::GlgP	2.5
PEP/PYR	p19	1.45
PTSE	p28	1.2
PEP/PYR	p20	0.2
PEIIA	G_cAMP	0.1
All other arcs		0 or NA
